# Serine tRNAs compete to regulate the mRNA translation of serine-sensitive codons

**DOI:** 10.1126/sciadv.ady4521

**Published:** 2025-11-14

**Authors:** Veronica Costiniti, Wyatt C. Tran, Nandhini Rajesh Babu, Evgeny Kanshin, Beatrix Ueberheide, Alec C. Kimmelman, Robert S. Banh

**Affiliations:** ^1^ Department of Biochemistry and Molecular Pharmacology, New York University School of Medicine, New York, NY 10016, USA.; ^2^ Laura and Isaac Perlmutter Cancer Center, New York University Langone Medical Center, New York University, New York, NY 10016, USA.; ^3^ Proteomics Laboratory, Division of Advanced Research Technologies, New York University School of Medicine, New York, NY 10016, USA.; ^4^ Department of Neurology, New York University Langone Health, New York, NY 10016, USA.; ^5^ Department of Radiation Oncology, New York University Langone Health, New York, NY 10016, USA.

## Abstract

Differential mRNA translation efficiency (mTE) of codons is important in regulating protein synthesis and cellular states and can change in response to amino acid availability. While the mTE of codons is canonically associated with their corresponding transfer RNA (tRNA) isoacceptors, its regulation by amino acids in mammalian cells remains unexplored. We found that ELAC2, a 3′ tRNA maturation endonuclease, decreases the mTE of UC[C/U] serine (Ser) codons in response to Ser limitation. Ablation of ELAC2 restored UC[C/U] mTE but reduced the mTE of AG[U/C] Ser codons. Among the tRNA^Ser^ isoacceptors, tRNA^Ser(GCU)^ decreased the most in *ELAC2*-deficient cells. Unexpectedly, tRNA^Ser(GCU)^ delivery restored AG[U/C] mTE and reduced UC[C/U] mTE in *ELAC2*-deficient cells. Last, we deciphered the effects of Ser-sensitive codons on mRNA translation and the human proteome. Our study revealed that in response to Ser limitation, regulation of tRNA^Ser(GCU)^ levels fine-tune the mTE of UC[C/U] or AG[U/C] Ser-sensitive codons and shapes the proteome.

## Teaser

Serine tRNAs compete to regulate translation of serine-sensitive codons to reshape the proteome during serine limitation.

## INTRODUCTION

Cellular organisms dynamically fine-tune their proteome in response to ever-changing internal and external signals, ensuring precise regulation of cellular function and survival. This regulation involves a complex array of mechanisms, including transcription, splicing, posttranslational modifications, and degradation. Central to this process is mRNA translation, where the mRNA translation efficiency (mTE) of codons plays a critical role in controlling protein synthesis. Bacteria and lower eukaryotes exhibit differences in the mTE of codons relative to the concentration of complementary transfer RNAs (tRNAs) (*1*–*4*). In bacteria, these differences can be functionally relevant; for instance, they can exploit differential mTE of serine (Ser) codons to induce biofilm formation in response to Ser deprivation (*5*). Classical mTE is regulated by rare codons, which are translated more slowly due to lower levels of corresponding charged tRNA isoacceptors (i.e., tRNAs with different anticodons that accept the same amino acids). In addition, expression differences among isodecoders (i.e., tRNA genes that have the same anticodon) can influence the total tRNA pool, further affecting translation speed, protein folding, and protein levels (*6*–*13*). Differences in mTE of codons were observed when human cells were deprived of several amino acids, such as proline (Pro), arginine (Arg), or Ser (*2*–*4*, *13*).

Ser-auxotrophic human cells can decrease the mTE of two of the six Ser codons, UC[C/U], to selectively affect protein synthesis in response to Ser limitation (*2*). The decreased mTE of human UC[C/U] Ser codons was correlated with low levels of charged tRNA^Ser(AGA)^, the corresponding isoacceptor. However, the pathways that regulate the mTE of Ser codons upon Ser deprivation are still not clear. Bacteria require Ser for the mTE of four of six Ser codons (UCN) (*14*), demonstrating differential codon mTEs between bacteria and humans. In addition, human cells can decrease the mTE of CG[U/C] Arg codons in response to Arg deprivation, which was correlated with decreased tRNA^Arg(ACG)^ charging (*3*). Similarly, Pro starvation decreased tRNA^Pro^ charging and increased the ribosomal stalling on Pro codons (*4*). To date, differential mTE of codons across species is thought to be regulated by the abundance or charging of the corresponding tRNA isoacceptor. However, the regulatory mechanisms that control tRNA levels or differential charging and their role in modulating the mTE of codons in response to amino acids are not clear.

Maturation of tRNAs requires 5′ and 3′ processing by ribonuclease P (RNase P) and RNase Z [also known as ELAC2 (ElaC ribonuclease Z 2) in mammals] following transcription by RNA polymerase III, respectively (*15*–*18*). Nuclear RNase P is a ribonucleoprotein complex that cleaves the 5′ leader sequence of premature tRNAs (pre-tRNAs) in the nucleus, distinct from the one in the mitochondria. Whereas ELAC2 is an endonuclease involved in cleaving the 3′ trailer sequence of pre-tRNAs in both the nucleus (Nuc) and mitochondria (Mito) driven by the differential translation of two in-frame isoforms (*15*, *19*, *20*). In addition, mature tRNAs undergo various RNA modifications (e.g., pseudouridylation, methylation, N^6^-threonylcarbamoylation, etc.) by specific enzymes to regulate tRNA wobbling, mRNA decoding, tRNA stability, or tRNA structure (*21*). For example, the EKC/KEOPS (elongator complex/kinase, endopeptidase, and other proteins of small size) can modify adenosine at position 37 (A37) to N^6^-threonylcarbamoyladenosine (t^6^A37) of tRNAs^(NNU)^ that decode “ANN” codons to maintain accurate translation and structural stability of the tRNA (*22*–*24*). tRNA synthetases can recognize and charge specific tRNA isodecoders with their corresponding amino acid for mRNA translation. Aminoacylated (also known as “Charged”) tRNAs bind to EEF1A1 (eukaryotic translation elongation factor 1 alpha 1), a guanosine triphosphatase, and are delivered to the A site of the ribosome during mRNA translation elongation (*25*–*27*). tRNA pools can vary across different tissues, cell types, microenvironments, and cell states in multicellular organisms, highlighting the dynamic nature of tRNA pools in biology (*28*–*30*). However, the pathways that regulate tRNAs in response to amino acid–dependent regulation of the mTE of codons is not known.

Here, we elucidate the mechanisms that regulate the mTE of Ser-sensitive codons, UC[C/U], in human cells. We identified that ELAC2 and other tRNA-related processes are important to regulate the mTE of UC[C/U] in response to Ser limitation. In addition, increased nuclear localization of ELAC2 is important for maintaining Ser tRNA (tRNA^Ser^) pools during Ser deprivation, specifically tRNA^Ser(GCU)^, to decrease the mTE of UC[C/U] Ser codons. ELAC2 ablation lowered tRNA^Ser(GCU)^ levels and switched the Ser-sensitive codons from UC[C/U], which is decoded by tRNA^Ser(AGA)^, to AG[U/C] across the entire transcriptome. By comparing the newly synthesized proteins of cells with UC[C/U] or AG[U/C] sensitivities, we reveal the key parameters to predict the proteins that are most likely affected by the mTE of Ser-sensitive codons in Ser-poor conditions.

## RESULTS

### mTE regulation of human Ser codons

**Fig. 1.**
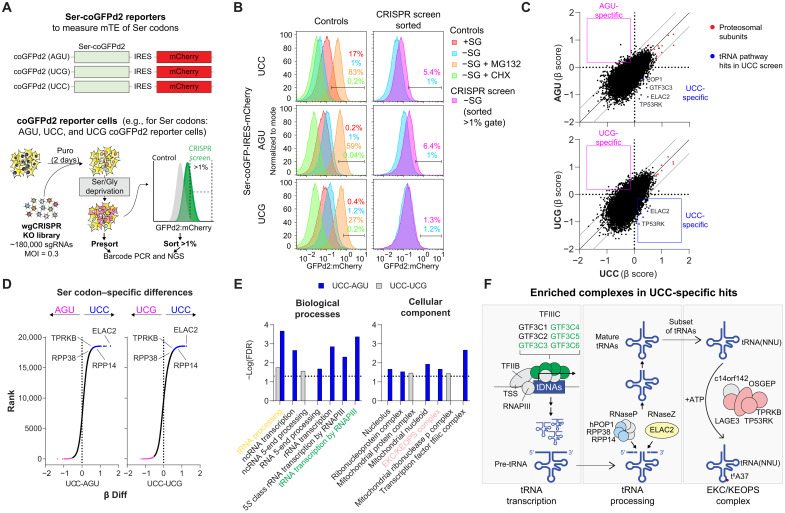
mTE regulators of Ser codons in response to Ser limitation. (**A**) Reporter screen to assess the mTE of Ser codons by CRISPR screening in Ser/Gly-deprived conditions. For each reporter, all Ser codons in Ser-coGFPd2 are encoded by one of the indicated Ser codons, followed by IRES-mCherry. A whole genome CRISPR (wgCRISPR) knockout (KO) screen was used to identify genes that regulate the mTE of Ser codons. A 1% control gate was used as a threshold to isolate enriched sgRNAs from cells. (**B**) Flow plots of each Ser-coGFPd2 reporter–expressing PATU-8902 cells grown [from (A)] with or without Ser/Gly (SG), MG132 (proteasomal inhibitor), and cycloheximide (CHX) for 24 hours. Ser/Gly-poor (−SG) samples were used to establish the sorting gate for cells. The screen was performed using two independent biological replicates for each Ser-coGFPd2 reporter line. Quantification of GFPd2:mCherry can be found in fig S1B. (**C**) Plots of the calculated β scores of genes from the CRISPR KO library in the indicated cells [from (B)]. Proteasomal subunits (red) served as common positive controls. UCC-specific hits are highlighted in blue, and AGU- or UCG-specific hits are shown in pink. (**D**) Ranked score of the β scores differences (β diff) from the CRISPR KO screen [from (C)] to identify Ser codon–specific hits. UCC-specific hits are highlighted in blue, while AGU- or UCG-specific hits are shown in pink. (**E**) GO enrichment of the biological processes (GO:BP) or cellular component (GO:CC) of the top ranking (>0.25) β diff scores for UCC [from (D)]. tRNA processing, transcription by RNA polymerase III (RNAPIII), and the EKC/KEOPS complex are highlighted. The dotted line represents the FDR = 0.05. (**F**) Top-ranking hits that regulate the mTE of UCC are enriched in the major processes that control tRNAs, as shown. MOI, multiplicity of infection; ncRNA, noncoding RNA; TFIIB, transcription factor IIB; TFIIIC, transcription factor IIIC; TSS, transcription start site; tDNAs, tRNA genes; ATP, adenosine triphosphate.

To elucidate the major mTE regulators of Ser codons, we used previously reported Ser codon–optimized destabilized green fluorescent protein (Ser-coGFPd2, ~2-hour half-life) reporters where all Ser codons were replaced with a specific Ser codon (e.g., UCC, UCG, and AGU) followed by internal ribosomal entry site (IRES)–mCherry (24-hour half-life) as an internal control (Fig. 1A) (*2*, *31*). These reporters served as a readout of the mTE of different Ser codons. To induce Ser deprivation, cells were also starved of Gly to prevent its potential conversion to Ser through serine hydroxymethyltransferase (*2*). The fluorescence of UCC (Ser-sensitive)–, but not AGU- and UCG (Ser-insensitive)–, coGFPd2 was the most decreased upon Ser/Gly deprivation, indicating lower mTE as previously reported (fig. S1A) (*2*).

A whole-genome CRISPR knockout (KO) screen (*32*) was used to identify genes that affect the mTE of specific Ser codons by increasing Ser-coGFPd2 fluorescence in Ser/Gly-deprived conditions (Fig. 1, A and B). Ablation of proteasomal subunits served as positive controls because GFPd2 is actively degraded by the proteasome (fig. S1, A and B). Consistent with this notion, proteasomal genes were found to be the highest hits in all Ser-coGFPd2 reporter cells (Fig. 1, B and C; fig. S1C; and table S1). Gene Ontology (GO) analysis of the positive hits [false discovery rate (FDR) < 0.25] further revealed differences in the genes that regulate the Ser-coGFPd2 reporters (fig. S1D). By calculating the difference between screens (*33*), we were able to identify Ser codon–specific regulators (Fig. 1D). While common hits (e.g., proteasomal genes) are not highly enriched in the differential scores between specific Ser codon screens (e.g., AGU versus UCC) (fig. S1C). Because UC[C/U] Ser codons exhibited the greatest mTE decrease in Ser-deprived conditions, we decided to focus on UCC-specific hits.

The screen did not identify genes in the major amino acid sensing pathways, mammalian target of rapamycin (mTOR), or general control nonderepressible 2 (GCN2), suggesting that the mTE regulation of specific Ser codons is independent of canonical amino acid sensing (table S1) (*34*). Instead, we found enrichment of genes involved in tRNA transcription, processing, and modification that regulated the mTE of UCC Ser codons upon Ser deprivation (Fig. 1, E and F). Transcription factor IIIC (e.g., GTF3C3-6) is critical for RNA polymerase III, pre-tRNA processing enzymes (e.g., *RPP14*, *ELAC2*, etc.) are important for the 5′ and 3′ maturation of tRNAs, and the EKC/KEOPS complex is needed to catalyze the N^6^-threonylcarbamoyladenosine (t^6^A) modification at position 37 (t^6^A37) on a subset of tRNAs that decode ANN codons (e.g., AG[U/C]) (Fig. 1F). Although tRNAs are important for mRNA translation, these tRNA processes have not been reported to regulate the mTE of specific codons.

### Ser codon preference is switchable

**Fig. 2.**
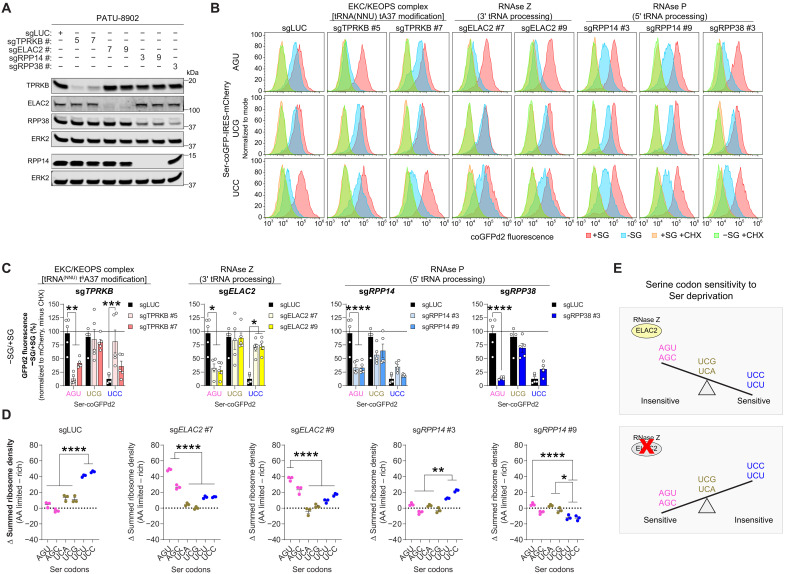
ELAC2 regulates Ser-sensitive codon preference in Ser-deprived cells. (**A**) Immunoblot of TPRKB, ELAC2, RPP38, and RPP14 in PATU-8902 cells to assess KO efficiency of sgRNAs. sgLUC (targeting luciferase) is used as a negative control. ERK2 serves as a loading control. (**B**) Representative flow plots of the indicated Ser-coGFPd2 reporter in PATU-8902 cells expressing sgRNAs targeting *TPRKB*, *ELAC2*, *RPP14* and *RPP38*. Ser-coGFPd2 reporter cells were grown with or without Ser/Gly (SG) or CHX for 24 hours. (**C**) Quantification of GFPd2 fluorescence ratios of Ser-coGFPd2 reporter cells grown in the absence and presence of Ser/Gly after 24 hours [from (B)]. Fluorescence intensities were normalized to mCherry, and CHX signal was subtracted to remove background signal (*n* = 5). (**D**) Ribosomal densities on Ser codons in mRNA under Ser/Gly-limiting conditions from Ribo-seq of PATU-8902 cells expressing sg*LUC* (control), sg*ELAC2*, or sg*RPP14*. The increased ribosomal density in Ser/Gly-poor conditions switches from UC[C/U] to AG[U/C] in ELAC2-deficient cells (*n* = 3). (**E**) Schematic of the type of Ser-sensitive codons in Ser/Gly-deprived conditions of control and *ELAC2*-KO cells. ELAC2 ablation switches from Ser-sensitive codons from UC[C/U] to AG[U/C]; where *n* represents the number of biologically independent replicates. Graphs (mean ± SEM) were compared using one- (D) or two-way (C) analysis of variance (ANOVA), followed by Bonferroni’s post hoc test (*P < 0.05, **P < 0.01, ***P < 0.005, and ****P < 0.001). AA, amino acid.

Next, we tested UCC-specific hits from the CRISPR screen on the mTE of Ser-coGFPd2 reporter cells in more detail (Fig. 2A). We evaluated the effects of several KOs on cellular proliferation and observed growth differences in Ser/Gly-rich conditions, but not under Ser/Gly deprivation (fig. S1E). Because differential mTE of Ser codons occurs specifically in Ser/Gly-poor environments where growth does not occur, these findings suggest that mTE changes are independent of differences in cell growth (fig. S1E).

We observed that *TPRKB*- (TP53RK binding protein), *ELAC2*-, *RPP14*- (ribonuclease P protein subunit 14), and *RPP38*- (ribonuclease P protein subunit 38) KO increased UCC-coGFPd2 fluorescence in Ser/Gly-poor conditions (Fig. 2B and figs. S1F and S2A). However, *RPP14*- and *RPP38*-KO also increased UCC-coGFPd2 fluorescence in Ser/Gly-rich conditions, demonstrating a nutrient-independent regulation of coGFPd2 levels (fig. S2A). The effects of *RPP14*- and *RPP38*-KO on UCC-coGFPd2 fluorescence upon Ser/Gly deprivation diminishes when normalized to Ser/Gly-rich conditions (Fig. 2C). While *TPRKB*- and *ELAC2*-KO significantly increased UCC-coGFPd2 fluorescence only in Ser/Gly-deprived conditions (Fig. 2, B and C, and fig. S2, A and B). This indicates that TPRKB and ELAC2 are important mTE regulators of UCC codons in response to Ser/Gly deprivation.

In contrast with UCC-coGFPd2, *TPRKB*-, *ELAC2*-, *RPP14*-, and *RPP38*-KO did not significantly affect the fluorescence of UCG-coGFPd2 in Ser/Gly-deprived conditions (Fig. 2, B and C). Although the ablation of RPP14 and RPP38 increased UCG-coGFPd2 fluorescence in Ser/Gly-rich and -poor conditions, there was no significant difference in fluorescence upon Ser/Gly deprivation when normalized to Ser/Gly-rich conditions (Fig. 2C and fig. S2B). The minimal effects observed here are consistent with the design of the screen in identifying UCC-specific genetic regulators.

Unexpectedly, we observed that *TPRKB*-, *ELAC2*-, and *RPP38*-, but not *RPP14*-, KOs decreased AGU-coGFPd2 fluorescence upon Ser/Gly deprivation (fig. S2A). In Ser/Gly-rich conditions, cells expressing *sgRPP14* and *sgRPP38*, but not *sgTPRKB* and *sgELAC2*, increased AGU-coGFPd2 fluorescence (fig. S2A). AGU-coGFPd2 fluorescence decreased for all genes upon Ser/Gly deprivation when normalized to Ser/Gly-rich conditions (Fig. 2C and fig. S2B). The ablation of TPRKB or ELAC2 specifically affects the mTE of Ser codons in Ser/Gly-deprived conditions, demonstrating that the mTE of UCC Ser codons can be regulated and that Ser codon preference can be changed between UCC and AGU Ser codons in a Ser-dependent manner.

### ELAC2 globally switches Ser codon preference

To assess the effects of the tRNA processing genes on the mTE of endogenous transcripts in Ser limiting condition, *ELAC2* and *RPP14* were selected for further analysis because they are required for 3′ and 5′ pre-tRNA processing, respectively. We performed ribosomal profiling (Ribo-seq) to measure the ribosome density upon Ser/Gly deprivation (*35*). Increased ribosomal density can reflect decreased mTE or stalling of the ribosome on specific codons (*2*–*4*). Consistent with our previous study (*2*), the ribosomal density was significantly increased on UC[C/U] Ser, but not other, codons upon Ser/Gly limitation in control cells (Fig. 2D and fig. S2C).

*ELAC2*- or *RPP14*-KO decreased the ribosomal density on UC[C/U] Ser codons in Ser/Gly-poor conditions compared to controls (Fig. 2D and fig. S2C). Whereas only *ELAC2*-KO significantly increased the ribosome density on AG[U/C] Ser codons across the entire transcriptome upon Ser/Gly limitation (Fig. 2D and fig. S2C). In addition, *ELAC2*- and *RPP14*-KO did not affect the ribosomal density on UCG and UCA (UC[G/A]) Ser codons or the other amino acid codons when deprived of Ser/Gly (fig. S2C). These observations are consistent with the Ser-coGFPd2 reporters. This indicates that ELAC2 can regulate the Ser-sensitive codon preference between UC[C/U] and AG[U/C] across the transcriptome in response to Ser/Gly-limiting conditions.

Because *ELAC2*- and *RPP14*-KO did not affect the ribosome density of codons compared to controls in Ser-rich conditions (fig. S2D), this indicated that defects in pre-tRNA processing enzymes do not cause global ribosomal stalling or decrease in mTE when Ser is available. Together, *RPP14*-KO can restore the ribosomal density and mTE of UC[C/U] Ser codons, but ELAC2 can specifically regulate the mTE of Ser codons by switching the Ser codon preference (e.g., UC[C/U] versus AG[U/C]) of human cells deprived of Ser, by unclear mechanisms (Fig. 2, D and E, and fig. S2E). Therefore, we decided to further study the role of ELAC2 in these processes.

### Nuclear ELAC2 localization regulates Ser codon preference

**Fig. 3.**
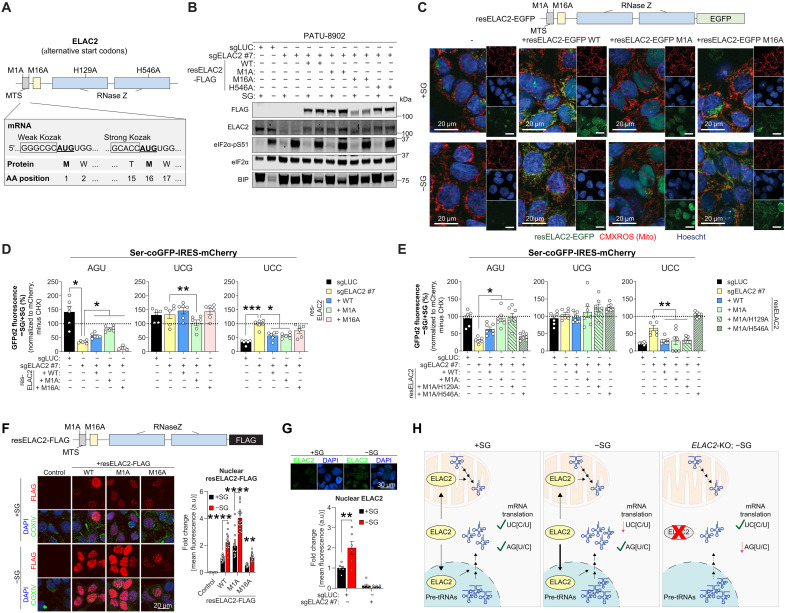
Nuclear ELAC2 regulates Ser-sensitive codons in Ser/Gly-deprived states. (**A**) Schematic of ELAC2 domains, signal sequences, and mutations used in this study. ELAC2 has two start codons, the first and second AUG contain weak and strong Kozak sequences, respectively. The RNase Z domain is important for ELAC2 catalytic activity and requires critical histidines (H129 and H546). (**B**) Immunoblots of PATU-8902 cells expressing sg*LUC* or sg*ELAC2* with or without sgRNA-resistant *ELAC2* (resELAC2) WT or mutants grown in Ser/Gly-rich or -poor media for 24 hours. eIF2α serves as a loading control. Note that exogenous expression of resELAC2 is lower than endogenous levels in cells. (**C**) Representative confocal fluorescent images of resELAC2–enhanced green fluorescent protein (EGFP) WT or mutants in PATU-8902 cells grown in Ser/Gly-rich or -poor environments. The mitochondria and nucleus were stained using MitoTracker DeepRed and Hoechst, respectively. Scale bar is shown. Enlarged EGFP images can be found in fig. S3C. (**D**) Quantification of corrected Ser-coGFPd2 fluorescence −SG/+SG ratios in the indicated PATU-8902 cells in response to Ser/Gly deprivation (*n* = 6). (**E**) Quantification of corrected Ser-coGFPd2 fluorescence (−SG/+SG) ratios to assess the contribution of the catalytic domain of nuclear resELAC2 (M1A) (*n* = 6). (**F**) Representative images and nuclear quantification of resELAC2-FLAG staining in PATU-8902 cells grown with or without Ser/Gly. The nucleus and mitochondria were stained using 4′,6-diamidino-2-phenylindole (DAPI) and cytochrome c oxidase subunit IV (COXIV). Scale bar is shown (*n* = 18). (**G**) Quantification and representative image of endogenous ELAC2 staining in PATU-8902 cells grown with or without Ser/Gly (*n* = 6). (**H**) Schematic illustrating how ELAC2 localization influences the mTE of UC[C/U] and AG[U/C] Ser codons by regulating pre-tRNA processing under Ser/Gly-deprived conditions (*n* = 6), where *n* represents the number of biologically independent replicates. Graphs (mean ± SEM) were compared by one- [(D) and (E)] or two-way ANOVA [(F) and (G)], followed by Bonferroni’s post hoc test (*P < 0.05, **P < 0.01, ***P < 0.005, and ****P < 0.001). a.u., arbitrary units.

The 5′ untranslated region (5′UTR) of ELAC2 harbors two in-frame start (AUG) codons: an upstream and downstream AUG with a weak and strong Kozak sequence, respectively (Fig. 3A) (*15*, *19*, *20*). Translation initiation on the first AUG (M1) codon produces full-length ELAC2 containing a mitochondrial targeting sequence (MTS) and nuclear localization signal. Initiation at the second AUG codon (M16) generates a truncated ELAC2 without an MTS, which only localizes to the nucleus (*15*). Under Ser/Gly-deprived conditions, endogenous ELAC2 protein levels decreased by ~25%, with a corresponding 50% reduction in mRNA levels (fig. S3, A and B), suggesting a transcriptional regulation. In addition, ELAC2’s RNase Z domain consists of an N-terminal and a C-terminal domains, with the latter playing a crucial catalytic role in pre-tRNA processing. The C-terminal RNase Z domain coordinates a Zn^2+^ ion to cleave pre-tRNAs, while the N-terminal domain lacks the ability to bind to Zn^2+^ and does not contribute directly to catalysis (*36*). While nuclear ELAC2 is expected to influence cytoplasmic mRNA translation, mitochondrial ELAC2 plays a critical role in mitochondrial translation and function (*20*). Therefore, it is not clear whether nuclear ELAC2 directly modulates the mTE of Ser codons or whether mitochondrial ELAC2 exerts indirect effects on cytoplasmic translation through mitochondrial dysfunction.

To elucidate the role of ELAC2 in regulating Ser codons preference in response to Ser/Gly deprivation, we expressed wild-type (WT), M1A (first AUG), or M16A (second AUG) single guide RNA (sgRNA)–resistant *5′UTR-ELAC2* cDNA (*resELAC2*) using the endogenous Kozak and cDNA sequences into *ELAC2*-KO cells (Fig. 3B). The M1A mutation prevents translation from the first start codon, bypassing the MTS and producing only nuclear ELAC2 (*15*). In contrast, the M16A mutation blocks translation from the second start codon, ensuring that only MTS-tagged ELAC2 is produced (*15*). The comparison of mitochondrial and nuclear ELAC2 is aimed at distinguishing direct nuclear tRNA processing effects from indirect impacts of mitochondrial dysfunction on translation or tRNA metabolism. resELAC2 M1A only localized to the nucleus, whereas most of the resELAC2 M16A entered the mitochondria (Fig. 3C and fig. S3C). Expression of resELAC2 WT restored Ser codon preference of *ELAC2*-KOs in Ser/Gly-deprived conditions as determined by UCC- and AGU-coGFPd2 fluorescence (Fig. 3D). Incomplete rescue of AGU-coGFPd2 by resELAC2 WT is reflected by lower exogenous ELAC2 levels compared to endogenous (Fig. 3B). This difference can be explained by the different activities or regulations of the native promoter and the cytomegalovirus promoter, which drives the *ELAC2* transgene. Loss of the MTS in resELAC2 M1A (i.e., nuclear ELAC2) significantly restored the mTE of AGU-coGFPd2 and suppressed the mTE of UCC-coGFPd2 in *ELAC2*-KO cells deprived of Ser/Gly (Fig. 3, B to D). Whereas resELAC2 M16A (i.e., mito-ELAC2) severely attenuated or weakly suppressed the fluorescence of AGU- or UCC-coGFPd2 in *ELAC2*-KO cells, respectively (Fig. 3, B to D). This suggests that either Mito:Nuc ELAC2 ratios are important for regulating Ser-sensitive codons or that the M16A mutation affects ELAC2 function. However, similar mTE differences were observed using codon-optimized ELAC2 and mutants, which strongly drives translation on the first AUG and disrupts the second Kozak sequence, in *ELAC2*-KO cells starved of Ser (fig. S3, D to F). This indicates that M16A mutation did not affect ELAC2 function and that the ratio of Mito:Nuc ELAC2 is important for the mTE regulation of Ser codons.

To investigate the role of ELAC2’s RNase Z domain in regulating Ser codon preference under Ser-limiting conditions, we mutated the Zn^2+^-coordinating histidine in the catalytic C-terminal domain (H546A) and introduced a similar mutation in the noncatalytic N-terminal domain (H129A). We found that the catalytically impaired H546A mutant prevented the rescue of WT and nuc-resELAC2 (M1A) in *ELAC2*-KO cells (Fig. 3E). Consistent with the inability to bind to Zn^2+^, the H129A did not affect the ability of WT and nuc-resELAC2 (M1A) to restore Ser codon preference in *ELAC2*-KO cells (Fig. 3E). Together, this indicates that ELAC2 catalytic activity is important for regulating the mTE of Ser codons. We also found that BIP/*HSPA5* (~80% UC[C/U] Ser codons) levels, previously shown to be affected by Ser/Gly deprivation (*2*), was rescued by *ELAC2*-KO and restored by WT and M1A resELAC2, but not M16A or catalytically inactive mutant (Fig. 3B). These data indicate that the catalytic activity of nuclear ELAC2 is important for regulating the mTE of Ser codons during the Ser/Gly limitation.

In response to Ser/Gly starvation, nuclear localization of resELAC2-FLAG WT and endogenous ELAC2 increased (Fig. 3, F and G). The ~15% rise in *ELAC2* transgene protein levels under Ser/Gly-poor conditions (Fig. 3B) likely contributes to the different expression patterns between endogenous and transgenic *ELAC2*. However, this modest increase alone is unlikely to account for the twofold increase in nuclear localization observed under Ser/Gly-poor conditions. Similarly, we also found that Ser/Gly limitation increased the levels of resELAC2-FLAG M1A mutant (Fig. 3, F and G). Although MTS-containing resELAC2 M16A exhibits lower nuclear localization in Ser/Gly-rich conditions, Ser/Gly deprivation also increased localization to the nucleus, albeit not to the extent of WT and M1A (Fig. 3, F and G). Therefore, Ser starvation increases ELAC2 localization into the nucleus to regulate the mTE of Ser codons in a manner that is dependent on the catalytic activity of the RNase Z domain, most likely to process pre-tRNAs^Ser^ (Fig. 3H).

### ELAC2 regulates the mTE of Ser codons via tRNA^Ser(GCU)^

**Fig. 4.**
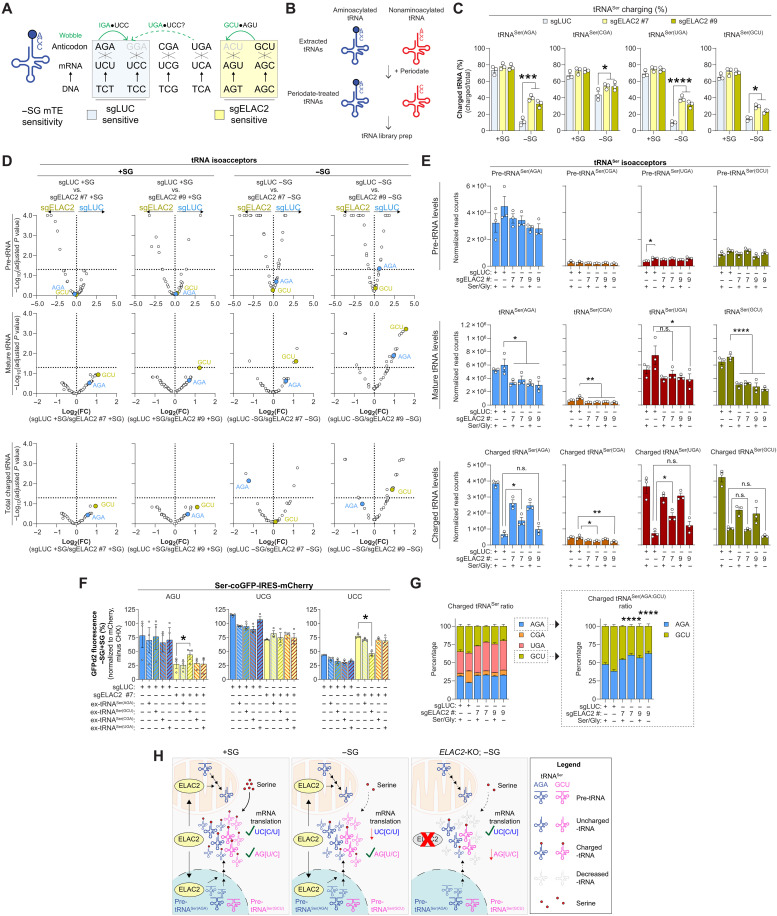
ELAC2 regulates the mTE of Ser codons via tRNA^Ser(GCU)^. (**A**) Schematic of Ser codon translation by tRNA^Ser^ isoacceptors, showing known (solid) and potential (dotted) wobbling pairing and inosine (“I”) modification. Ser-sensitive codons for control (sgLUC) and *ELAC2*-KO cells are highlighted. (**B**) Diagram of charged tRNA-seq workflow to measure charged and uncharged tRNAs. (**C**) tRNA^Ser^ charging in PATU-8902 control or *ELAC2*-KO cells grown with or without Ser/Gly for 24 hours (*n* = 3). (**D**) Premature (pre)-, mature, and charged tRNA isoacceptor levels in PATU-8902 control and *ELAC2*-KO cells grown with or without Ser/Gly (SG) for 24 hours. Dotted line indicates adjusted *P* value = 0.05 (*n* = 3). (**E**) Differences of pre-, mature, and charged tRNA^Ser^ isoacceptors in the indicated PATU-8902 cells grown with or without SG for 24 hours (*n* = 3). (**F**) Ser-coGFPd2 fluorescence ratio of PATU-8902 control and *ELAC2*-KO cells transfected with exogenous (ex)–tRNA^Ser^ isodecoders grown in Ser/Gly-rich and -poor conditions for 24 hours (*n* = 4). (**G**) Charged tRNA^Ser^ isoacceptors ratios in PATU-8902 control and *ELAC2*-KO cells grown with or without Ser/Gly for 24 hours. Isolated comparison of tRNA^Ser(AGA)^ and tRNA^Ser(GCU)^ ratios on the right (*n* = 3). (**H**) Schematic of nuclear ELAC2 in modulating charged tRNA^Ser(GCU)^ and tRNA^Ser(AGA)^ to regulate mTE of Ser codons. In Ser/Gly-rich environments, tRNA^Ser^ are fully charged, supporting translation of both UC[C/U] and AG[U/C]. Ser/Gly deprivation reduces charged tRNA^Ser(AGA)^ and tRNA^Ser(GCU)^, lowering mTE of UC[C/U] Ser codons. ELAC2 ablation decreases tRNA^Ser(GCU)^ (~3-fold) and tRNA^Ser(AGA)^ (~2-fold), alters charged tRNA^Ser^ ratios, and decreases mTE of AG[U/C] in Ser/Gly-deprived conditions. Arrow direction indicates higher protein abundance in control or *ELAC2*-KO cells; where *n* represents biologically independent experiments. Graphs (mean ± SEM) were compared by one- [(E) and (F)] or two-way [(C) and (D)] ANOVA, followed by Dunnett’s [(E) and (F)], Tukey’s (C) post hoc test, or two-stage Benjamini-Hochberg correction (D). (*P < 0.05, **P < 0.01, ***P < 0.005, and ****P < 0.0001). FC, fold change; n.s., not significant.

There are four major tRNA^Ser^ isoacceptors, which contain AGA, CGA, UGA, or GCU anticodon sequences and decode the six Ser codons during mRNA translation (Fig. 4A). Because tRNA^Ser(AGA)^ is modified at position A34 to inosine (I34), it can decode both UCC and UCU Ser codons (*37*, *38*). Similarly, tRNA^Ser(GCU)^ can decode both AGU and AGC Ser codons due to tRNA wobbling (*21*). Because nuclear ELAC2 catalytic activity on 3′ processing of pre-tRNAs was required to regulate the mTE of Ser codons, we assessed the effects of *ELAC2*-KO on tRNA^Ser^ charging and levels using charged tRNA sequencing (tRNA-seq; Fig. 4B) (*2*, *39*). As expected, the charging of tRNA^Ser^ isoacceptors decreased upon Ser/Gly starvation (Fig. 4C and fig. S4A).

Because *ELAC2* ablation switches the Ser-sensitive codons from UC[C/U] to AG[U/C], we predicted that there may be reciprocal changes in the corresponding tRNAs, tRNA^Ser(AGA)^ and tRNA^Ser(GCU)^, respectively. Consistent with the role of ELAC2 in pre-tRNA processing, *ELAC2*-KO cells had lower levels of several mature tRNAs and higher levels of some pre-tRNAs (Fig. 4D and fig. S4B). Ser/Gly deprivation did not alter pre-tRNA or mature tRNA isoacceptor levels but significantly affected isodecoder expression (fig. S4, C and D), suggesting regulation at the level of specific tRNA genes. We observed that *ELAC2*-KO in Ser/Gly-poor conditions significantly reduced tRNA^Ser(GCU)^ (2.2- to 3-fold) more than tRNA^Ser(AGA)^ (1.5- to 2-fold) isoacceptors (fig. S4B). In line with this, *ELAC2*-KO also differentially affected specific tRNA^Ser^ isodecoders (fig. S4, E and F), further supporting the idea of isodecoder-specific regulation. Whereas the levels of charged tRNA^Ser^ were not consistently affected in *ELAC2*-KO cells deprived of Ser/Gly (Fig. 4E and fig. S4F). Neither the percentage of tRNA^Ser^ charging, tRNA^Ser^ levels, nor total charged tRNA^Ser^ levels were correlated with the ribosomal stalling observed in both the control and *ELAC2*-deficient cells (fig. S4, G to I). These data suggest that only one of the changes in tRNA^Ser(AGA)^ or tRNA^Ser(GCU)^ is responsible for driving Ser codon preference in control or *ELAC2*-KO cells under Ser/Gly deprivation.

To bypass the need of ELAC2 for 3′ processing of pre-tRNAs, we transfected exogenous mature tRNAs (ex-tRNAs) to increase intracellular levels of specific tRNA^Ser^ isodecoders in control and *ELAC2*-KO cells (*40*). ex-tRNA^Ser^ isoacceptors did not affect the mTE of Ser codons in control or *ELAC2*-KO cells in Ser/Gly-rich conditions (Fig. 4F). ex-tRNA^Ser^ isoacceptors also did not affect the mTE of Ser codons in control cells under Ser/Gly deprivation, suggesting that increasing tRNA^Ser^ alone is insufficient to alter Ser-sensitive codons in control cells (Fig. 4F). Unexpectedly, ex-tRNA^Ser(GCU)^, but not ex-tRNA^Ser(AGA, CGA, UGA)^, isoacceptor restored both the mTE of AG[U/C] and UC[C/U] Ser codons in *ELAC2*-KO cells deprived of Ser/Gly (Fig. 4F). The lack of effect from tRNA^Ser(AGA)^ transfection on the mTE of UC[C/U] codons under Ser/Gly-deprived conditions does not rule out its potential importance but may instead reflect technical limitations such as suboptimal tRNA delivery into the cytoplasm or the absence of critical posttranscriptional modifications. Because ex-tRNA^Ser(GCU)^ delivery restored the mTE of Ser codons in *ELAC2*-KO cells, we investigated whether endogenous tRNA pools were affected. While the total ratios of tRNA^Ser^ isoacceptors remained relatively stable (fig. S4J), the ratios of charged tRNA^Ser^ isoacceptors were specifically altered in *ELAC2*-KO cells under Ser/Gly-deprived conditions (Fig. 4G). A direct comparison between tRNA^Ser(AGA)^ and tRNA^Ser(GCU)^ revealed a similar shift (Fig. 4G), suggesting that the combination of Ser limitation and ELAC2 loss selectively affects the charged tRNA pool. Together, these results indicate that ELAC2 regulates Ser-sensitive codons by maintaining charged tRNA^Ser^ isoacceptor pools during Ser/Gly deprivation (Fig. 4H).

### Ser codon preference affects the proteome in Ser-limiting conditions

**Fig. 5.**
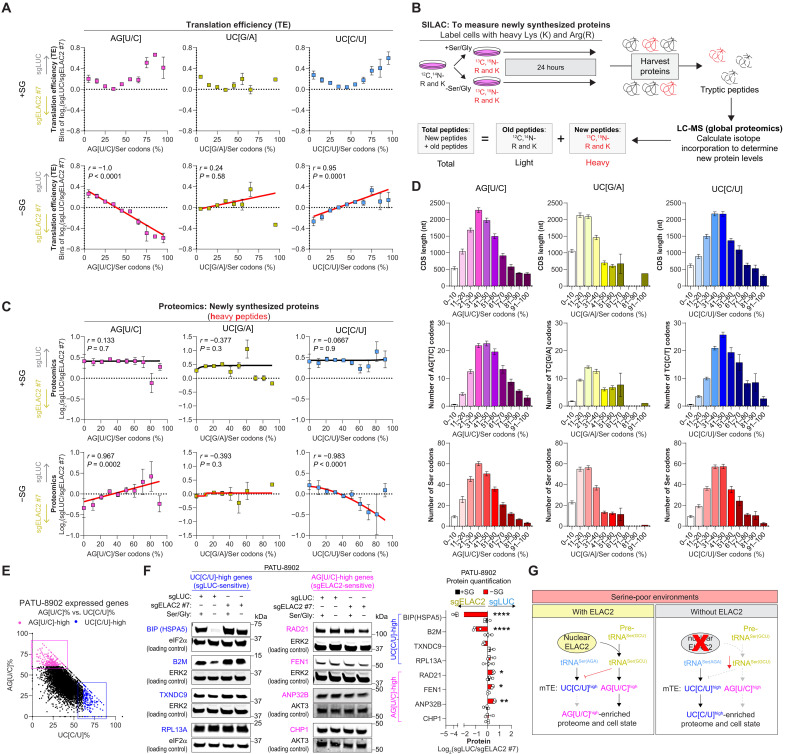
Ser-sensitive codon preference affects the proteome in response to Ser/Gly limitation. (**A**) Differences in TE in PATU-8902 control and *ELAC2*-KO cells grown with and without Ser/Gly for 24 hours plotted against the percentage bin of the indicated Ser codons. (**B**) SILAC schematic to measure newly translated proteins after 24 hours in Ser/Gly-rich or -poor conditions. Newly synthesized peptides are labeled with ^13^C, ^15^N-Arg (R), and -Lys (K), while old existing peptides remain unlabeled. (**C**) Fold change of newly synthesized proteins (heavy labeled) in PATU-8902 control and *ELAC2*-KO cells grown with or without Ser/Gly for 24 hours relative to the percentage bin of the indicated Ser codon. Nonlinear regression lines excluded outliers, typically in the 91 to 100% bin (*n* = 3). (**D**) Histogram of the CDS length, number of specific, or total number of Ser codons of proteins [from (C)] relative to the percentage bin of the indicated Ser codons (*n* = 3). (**E**) Scatterplot of the percentage of AG[U/C] and UC[C/U] Ser codons for genes expressed in PATU-8902 cells. Genes that are 61 to 90% AG[U/C] or UC[C/U] are highlighted as likely sensitive to reduced mTE. (**F**) Immunoblots of UC[C/U] (blue)– and AG[U/C] (pink)–high genes in PATU-8902 control and *ELAC2*-KO cells grown in Ser/Gly-rich and -poor conditions for 24 hours. ERK2, eIF2α, and AKT3 serve as loading controls (left). Quantification of ELAC2-dependent protein changes of UC[C/U]- and AG[U/C]-high genes in Ser/Gly-replete or -deprived conditions (right) (*n* = 3). (**G**) Schematic of ELAC2-dependent regulation of tRNA^Ser(GCU)^, the mTE of Ser-sensitive codons, and the effects on the proteome under Ser-deprived conditions. Arrow direction indicates higher protein abundance in control or *ELAC2*-KO cells; where *n* is displayed as individual points and represents biologically independent samples. Graphs (mean ± SEM) were compared. Nonlinear regression lines were fitted, and Spearman correlation coefficients are represented by *r* (*P < 0.05, **P < 0.01, and ****P < 0.0001).

Prior studies did not find a strong correlation between the total proteome and the percentage or number of Ser-sensitive codons or length of genes (*2*). In Ser/Gly-deprived conditions, we observed a weak correlation between differential translation efficiency (TE) values from Ribo-seq and RNA sequencing (RNA-seq) datasets when genes were analyzed individually based on the percentage of specific Ser codons, UC[C/U], AG[U/C], and UC[G/A] (fig. S5A) (*41*). We hypothesized that binning genes by the relative proportion of UC[C/U], AG[U/C], and UC[G/A] codons over total Ser codons would reduce noise from gene-specific regulatory effects and better capture global trends in translational regulation. We observed a high correlation between the differential TEs when binned into different percentages of Ser codons. Supporting this, we found a strong correlation between differential TE values when genes were binned by codon percentages (Fig. 5A). Higher differential mTE scores were associated with ribosome stalling–prone Ser codons: UC[C/U] in sg*LUC* (control) cells and AG[U/C] in *ELAC2*-KO cells (Fig. 5A and fig. S5A). This observation provided a potential parameter for evaluating the effects of Ser-sensitive codons across the proteome.

Other than mRNA translation, proteins also can be regulated by degradation (e.g., proteasome and lysosome), splicing, transcription, or UTRs, thereby making it difficult to isolate the effects of Ser-sensitive codons on the proteome. To overcome these issues, we used SILAC (stable isotope labeling of amino acids in cell culture) to measure newly synthesized proteins to determine the effects of altered Ser codon preference on the proteome (Fig. 5B) (*42*). We first assessed the predictive value of the absolute number of Ser-sensitive codons and the previously reported *z*-score of UC[C/U]:UC[G/A] codons across genes. Neither of the two analyses found strong correlation with either total or newly synthesized protein levels in Ser/Gly-poor conditions, whether analyzed individually or by binning them (fig. S5, B and C) (*2*). Poor associations by the absolute number of Ser-sensitive codons may be attributed to confounding factors, such as differences in coding sequence (CDS) length or the fact that the absolute number of Ser-sensitive codons does not account for the total number of Ser codons or the presence of Ser-insensitive codons within each gene. This would suggest that relying solely on the absolute number of Ser-sensitive codons may be insufficient and that some form of normalization is necessary. While the original *z*-score did not account for AG[U/C] codons, which we identified as Ser sensitive in *ELAC2*-KO cells. In contrast, the *z*-score of AG[U/C]:UC[C/U] codons showed a stronger correlation with newly synthesized proteins when genes were binned by *z*-score (*r* = 0.75), compared to a weak correlation when analyzed individually (*r* = 0.076) under Ser/Gly-deprived conditions (fig. S5, B and C). These findings suggest that codon composition, particularly the balance of Ser-sensitive and -insensitive codons, plays a key role in shaping translational output and the proteome.

We also examined the relationship between differential TE values and newly synthesized protein levels, finding only weak correlations both at the individual gene level (*r* = −0.02) and after binning (*r* = −0.54) (fig. S5D). These results suggest that differential TE alone is not as predictive of translational output as codon composition in Ser/Gly-deprived conditions. When proteins were plotted individually, neither total nor newly synthesized proteins appeared to correlate with the percentage of Ser-sensitive codons between control and *ELAC2*-KO cells (fig. S5E). This is likely due to noise of multiple modalities that can regulate protein levels.

Given that differential TE values showed strong correlations when genes were binned by the proportion of UC[C/U], AG[U/C], and UC[G/A] codons (Fig. 5A), we applied the same binning approach to evaluating how Ser-sensitive codon composition influences the proteome (Fig. 5C and table S2). Consistent with the lack of ribosomal stalling in Ser-rich conditions, no correlation was observed for newly synthesized proteins between control and *ELAC2*-KO cells in any of the three Ser codon clusters (Fig. 5C). While in Ser/Gly-poor conditions, newly synthesized proteins with 61 to 90% AG[U/C] and UC[C/U] Ser codons were more strongly enriched in controls (i.e., UC[C/U]-sensitive) and *ELAC2*-KO (i.e., AG[U/C]-sensitive) cells, respectively (Fig. 5C). As expected, the percentage of UC[G/A] was not correlated with either the control or *ELAC2*-KO proteome. Similar, but weaker, patterns were observed in the total proteome (fig. S5F). However, proteins with 91 to 100% AG[U/C] or UC[C/U] Ser codons did not follow the same trend (Fig. 5C and fig. S5F). This suggests that the percentage of Ser-sensitive codons alone does not completely predict effects on the proteome.

To understand the nonconformity of the 91 to 100% bin by proteomics, we assessed the CDS length, number of specific Ser codons, and total Ser codons for all genes binned in their clusters (Fig. 5D). A Gaussian distribution was observed for all parameters in the AG[U/C] and UC[C/U] clusters. Whereas binning by UC[G/A] showed a left-skewed distribution. The 91 to 100% AG[U/C] and UC[C/U] bins were the shortest CDS with the lowest number of sensitive and total Ser codons (Fig. 5D). This suggests that the handful of genes with 91 to 100% Ser-sensitive codons are not affected because of their short gene length or low number of Ser-sensitive codons. Changes in mRNA levels also could account for the altered protein differences of genes with 91 to 100% UC[C/U] codons, but not for those with 91 to 100% AG[U/C] codons (fig. S5G). In addition, the correlation differences between the differential TE values (Fig. 5A) and newly synthesized proteins (Fig. 5C) for genes with >90% of a Ser codon type suggest that posttranslational mechanisms may also regulate the abundance of these proteins.

Together, these data reveal that the proteins most likely affected by Ser/Gly deprivation are genes that are encoded with 61 to 90% Ser-sensitive codons (Fig. 5C). Molecular function analysis of AG[U/C]- or UC[C/U]-enriched genes reveal differences in proteins that may be affected by the mTE of Ser-sensitive codons (Fig. 5E, fig. S5H, and table S3). Some chaperones and ribosomal RNA (rRNA) binding proteins are UC[C/U] enriched, such as *HSPA5* (also known as BIP; Fig. 5F, figs. S3B and S5H, and table S3). Whereas several nucleases are AG[U/C] enriched (Fig. 5F, fig. S5H, and table S3). To further investigate this list, we examined how *ELAC2*-KO affects protein levels of several UC[C/U]- and AG[U/C]-rich genes under Ser/Gly-rich or -poor conditions. Specifically, *ELAC2*-KO cells, which exhibit impaired translation of AG[U/C] codons, showed higher levels of UC[C/U]-rich proteins (HSPA5, B2M, and TXNDC9). In contrast, control cells, which are more sensitive to UC[C/U] codons, displayed higher levels of AG[U/C]-rich proteins (RAD21, FEN1, and ANP32B) (Fig. 5F), despite similar mRNA levels between control and *ELAC2*-KO cells in Ser/Gly-deprived conditions (fig. S5I). Some AG[U/C]- (e.g., CHP1) or UC[C/U]- (e.g., RPL13A) rich genes did not show expected differences in total protein levels, likely due to differences in protein stability or degradation. In addition, although ELAC2 ablation increased the expression of several transcripts under Ser/Gly-rich conditions, the corresponding protein levels did not show a significant change (Fig. 5F and fig. S5I). Therefore, it is possible that mTE differences between Ser-sensitive codons can affect the proteome and can affect the cell state or response in Ser/Gly-limiting conditions (Fig. 5G).

## DISCUSSION

We previously reported that decreased mTE of UC[C/U] Ser codons in Ser-limiting environments was correlated with charged tRNA^Ser(AGA)^ levels (*2*). Our study to identify the mTE regulators of UC[C/U] demonstrates that the abundance of charged tRNA^Ser(AGA)^ cannot explain the switch of Ser sensitivity to AG[U/C] codons upon *ELAC2* deletion. Unexpectedly, changes in tRNA^Ser(GCU)^ levels alone were sufficient to regulate the mTE of UC[C/U] or AG[U/C] in Ser-deprived conditions (Fig. 4, F to H). This is consistent with the similar effects observed for the perturbation of the EKC/KEOPS complex (e.g., sg*TPRKB*) on the mTE of UCC- and AGU-coGFPd2 reporters (Fig. 2C). Deficiencies of the EKC/KEOPS complex would decrease the N^6^-threonylcarbamoyl modification of position A37 (t^6^A37) of ANN-decoding tRNAs, such as tRNA^Ser(GCU)^ for AG[U/C] codons. tRNA^Ser(GCU)^ is reported to be modified on position A37 with N^6^-methyl-N^6^-threonylcarbamoyl (m^6^t^6^A37), which requires the initial modification by EKC/KEOPS (*43*, *44*). On the basis of the role of t^6^A37, decreased t^6^A37 modifications may decrease tRNA^Ser(GCU)^ levels, charging, or decoding efficiency (*22*–*24*) to allow switching of Ser-sensitive codons in response to Ser deprivation. These data argue that under poor tRNA^Ser^ charging conditions by Ser limitation, competition between tRNA^Ser(GCU)^ and tRNA^Ser(AGA)^ exists to regulate the mTE of AG[U/C] and UC[C/U] Ser codons independently of the other tRNA^Ser^ isoacceptors (i.e., CGA and UGA).

In 2003, Elf *et al*. (*12*) proposed a model in *Escherichia coli* predicting that, during amino acid starvation, codons whose translation rate is insensitive to amino acid depletion are preferentially used for translation. This is driven by the selective charging of specific tRNA isoacceptors, which favors certain codons under amino acid–limiting conditions. This model was tested by experimental studies in bacteria (*11*, *45*) and extended to mammalian cells (*3*, *13*). In bacteria, Ser deprivation reduces translation of four of the six Ser codons (e.g., UCN), hinting at potential codon-specific differences in mTE in mammalian cells (*5*, *45*). In human embryonic kidney (HEK) 293 cells, Saikia *et al*. (*13*) reported that amino acid starvation decreased ribosome-associated tRNA^Ser(AGA, GCU)^ and increased ribosome-associated tRNA^Ser(CGA, UGA)^, suggesting that translation of UC[C/U] and AG[U/C] Ser codons is particularly sensitive to Ser availability. However, HEK293 cells have an active Ser biosynthesis pathway (*46*), which can suppress Ser codon–specific mTE differences under complete Ser deprivation in mammalian cells (*2*). Our findings expand and refine the proposed model by introducing layers of insight. Similar to recent studies (*2*, *3*, *5*, *45*), our model focuses on the impact of depleting a single amino acid to investigate codon bias. This approach allowed us to detect subtle differences that the broader model might overlook. By analyzing differential scores between specific Ser codons (e.g., AGU versus UCC) from our CRISPR screen, we uncovered a regulatory mechanism that adds depth to our understanding of Ser codon bias. In our study, *ELAC2*-KO shifted the Ser-sensitive codons from UC[C/U] to AG[U/C], yet the percentage of tRNA charging increased for both tRNA^Ser(AGA)^ and tRNA^Ser(GCU)^ under Ser/Gly-deprived conditions. This suggests that percent tRNA charging alone does not fully explain the differences in mTE between Ser codons in mammalian cells. Instead, differences in the absolute pools of charged tRNA^Ser^ isoacceptors may drive selective translation, potentially through competition for EEF1A1-mediated delivery to the ribosome (*47*, *48*). This suggests that beyond the established model of selective tRNA charging, (*12*), tRNA competition or additional regulatory mechanisms contribute to shaping the mTE differences of Ser codons in Ser/Gly-deprived mammalian cells. Further investigation is required to determine whether other codons similarly use multiple tRNA regulatory mechanisms to modulate their mTE in response to specific amino acid–deprived conditions.

In our model, percentage tRNA charging is not largely different between tRNA^Ser(GCU)^ and tRNA^Ser(AGA)^, suggesting that competition is unlikely caused by seryl-tRNA synthetase (SARS1). Instead of SARS1, competition may exist during the delivery of tRNA^Ser(GCU)^ and tRNA^Ser(AGA)^ to the ribosome by EEF1A1. Although tRNA^Ser(AGA)^ and tRNA^Ser(GCU)^ are highly similar, they mostly differ in their variable arm and T-stem sequences that are required to bind to Ser-tRNA synthetase (SARS1) and EEF1A1, respectively (fig. S5J). Other studies have shown that the tRNA binding strength of bacterial EEF1A1 homolog, EF-Tu, depends on the specific T-stem base pairs at position 49-65, 50-64, and 51-63 and predicted stronger affinity for tRNA^Ser(GCU)^ than tRNA^Ser(AGA)^ (*47*, *48*). The binding strength of EEF1A1 to charged tRNAs depends on the specific amino acid and the T-stem of tRNA, more specifically at base pairs 49-65, 50-64, and 51-63 (*25*–*27*). Because tRNA^Ser(GCU)^ and tRNA^Ser(AGA)^ isodecoders carry the same amino acid and differ at the all three T-stem positions (fig. S5J) (*49*), the mTE of Ser-sensitive codons is likely regulated by competition for EEF1A1 and delivery to the ribosome during translation. Further work characterizing this hypothesis is needed in the future. Unlike the canonical correlations of decrease mTE of codons with levels of their respective tRNA isodecoder (*2*–*4*), we report that decrease mTE of human UC[C/U] is controlled by the levels of a noncorresponding isoacceptor, tRNA^Ser(GCU)^. Whether similar tRNA competition exists to regulate the mTE of other codons, such as CG[U/C] Arg codons (*3*), remains to be elucidated.

Recently, triple KO of *METTL2A/2B/6* was reported to decrease position C32 methylation on tRNA^Ser(GCU)^, increase ribosomal stalling on AGU Ser codons in Ser-rich conditions, decrease AGU-rich DNA damage repair proteins, and increase sensitivity to cisplatin (*50*). We did not identify METTL2A/2B/6 in our screen due to the nonredundant function of the paralogs. In addition, neither the ablation of the KEOPS complex nor *ELAC2* affected the mTE of Ser codons in Ser-rich conditions (fig. S2D). This would argue for a mechanism, where altered charged tRNA levels, and not only modification-dependent properties, regulate the Ser codon preference in Ser-limiting conditions. Unexpectedly, we did not identify genes involved in major amino acid sensing (e.g., mTOR or GCN2) (*51*–*54*) or ribosomal collision sensing (e.g., ZAK1, RNF14, RNF25, GCN1, etc.) (*55*–*59*) pathways. It is possible that these pathways are critical for survival under Ser-deprived conditions, or it suggests that ribosomal collisions are not highly occurring upon Ser/Gly limitation. As Ser/Gly limitation decreases tRNA^Ser^ charging, GCN2 would phosphorylate eIF2α, which would reduce mRNA translation initiation and lower the chances of ribosomal collisions. Therefore, stress response pathways that increase phosphorylation of eIF2α, such as GCN2, PERK, PKR, and HRI (*60*), to decrease mRNA translation initiation also may reduce the risk of ribosomal collisions. Consistent with this notion, ribosomal collision pathways were more strongly activated in glutamine-starved cells treated with ISRIB (integrated stress response inhibitor), which blocks the translation-slowing effects of eIF2α phosphorylation (*59*). It remains unclear whether the same holds true under Ser/Gly-deprived conditions. Therefore, it is still possible that our screen either missed or was not sensitive enough to detect amino acid sensing or ribosome collision pathway genes as Ser codon–specific hits. Nonetheless, our study found that Ser/Gly limitation can decrease the mTE of UC[C/U] or AG[U/C] by regulating the levels of cytoplasmic tRNA^Ser(GCU)^.

ELAC2 is the only known 3′ pre-tRNA processing enzyme in mammals. However, we found that ELAC2 ablation did not affect the levels of all tRNAs equally, suggesting other mechanisms that regulate tRNAs. *ELAC2*-KO led to decreased levels of a subset of specific mature tRNAs in both Ser/Gly-rich and -poor conditions, including Arg (CCU), His (GUG), Leu (CAG, UAA, and UAG), Pro (AGG, CGG, and UGG), SeC (UCA), Ser (AGA, CGA, and GCU), and iMet (CAU) (fig. S4B). The basis for these selective changes remains unclear but may involve a complex interplay between tRNA stability, processing efficiency, or degradation dynamics. This will be important to explore further in future work. It also is possible that there are other nucleases with different substrate specificities involved in the 3′ processing of pre-tRNAs or differences in tRNA half-lives in mammalian cells. ELAC1 is a paralog of ELAC2 and has been shown to repair 3′ damage on tRNAs and only cleaves pre-tRNAs with very short 3′ trailers (2 to 5 nt) (*61*). In addition to RNase Z, bacteria also can use ribonucleases such as RNase E, RNase PH, and RNase T (*62*, *63*). Whereas yeast can also use 3′ exonucleases, Rex1p and Rrp6p, for 3′ end pre-tRNA maturation (*64*, *65*). Therefore, it is possible that additional nucleases act to compensate or process a distinct pool of pre-tRNAs in the absence of ELAC2 in the nucleus. Further work characterizing the increased nuclear localization of ELAC2, either by understanding the differential translation initiation or by posttranslational modifications, would illuminate the regulation of ELAC2 in the dynamic regulation of pre-tRNA processing and maturation. Whether dynamic regulation or localization of ELAC2 exists in response to other stimuli in mammals remains to be elucidated.

One long-standing question remains: What is the biological or evolutionary importance, if any, of selective mRNA translation of Ser-sensitive codons during human development? *Bacillus subtilis* relies on differential mTE of Ser codons to induce biofilm formation in response to Ser depletion in the media (*5*). Therefore, the role of mTE differences of Ser codons in mammalian biology will likely rely on the conditions or environments that have low Ser levels, such as the brain and dietary deficiencies, liver disease, renal disease, or metabolic disorders. Ser is a conditionally essential amino acid that is obtained from our diets or can be synthesized by the liver, kidney, and other cells. However, not all cell types can up-regulate Ser biosynthesis in response to Ser deprivation. Tumors can outgrow their blood supply and develop areas of nutrient scarcity, such as Ser. In response, the decreased mTE of UC[C/U] Ser codons can allow the selective translation of UC[C/U]-poor genes, such as nerve growth factor, into the tumor (*2*). UC[C/U] codons are enriched in many olfactory receptors and G-coupled protein (e.g., *DRD1*, *OR2A2*, etc.) genes that are expressed in the olfactory bulbs and brains of mammals (*66*). Differential mTE of UC[C/U] may play a key role in maintaining receptor levels in cells. Although pathogenic biallelic ELAC2 mutations in patients are associated with psychomotor delay, hypotonia, and hypertrophic cardiomyopathy (*67*, *68*), it is not clear which phenotypes are driven by mitochondrial dysfunction or defects in maintaining specific nuclear tRNA isoacceptor pools. Therefore, the importance and roles of differential mTE of Ser, and potentially other, codons in response to amino acid status in humans biology and disease remains an important area for future investigation.

## MATERIALS AND METHODS

### Cell lines

PATU-8902, HEC-1-A, and HEK293T cell lines were purchased from the American Type Culture Collection and Deutsche Sammlung von Mikroorganismen und Zellkulturen. Cell lines were maintained in Dulbecco’s modified Eagle’s medium (DMEM) + 10% FBS (fetal bovine serum) + 1% penicillin/streptomycin. None of the cell lines used in this study are found in the International Cell Line Authentication Committee or National Center for Biotechnology Information Biosample databases of commonly misidentified cell lines. Cell lines were routinely verified to be negative for mycoplasma by polymerase chain reaction (PCR). Cell lines were authenticated by periodic fingerprinting and visual inspection, and low passage cultures were carefully maintained in a central laboratory cell bank as described previously (*2*). Where indicated, cells were infected with lentivirus-expressing specific cDNAs, sgRNAs, and Cas9 and selected with puromycin (2 μg/ml) and/or blasticidin (10 μg/ml), respectively. All cell lines were grown and maintained in DMEM + 10%FBS at 37°C in a humidified atmosphere of 95% air and 5% CO_2_.

### Reagents

Chemicals and antibodies were purchased at the specified manufacturer. Antibodies were used for immunoblotting or immunostaining at manufacturer-recommended concentrations. See table S4 for the complete list of reagents used in this study.

### Whole genome sgRNA CRISPR screen

Whole genome CRISPR KO positive selection screen was performed using an all-in-one library containing ~187,535 sgRNAs targeting 18,663 genes (~10 guides each) with 1000 nontargeting and 500 intergenic sgRNA controls as described (*32*). PATU-8902 Ser-coGFPd2 reporter cells were infected with a multiplicity of infection of <0.3 to obtain 100× coverage of the library, followed by selection in puromycin (2 μg/ml) for 48 hours. After 7 days postinfection, cells were seeded and starved in Ser/Gly-deprived media for 24 hours. Control cells were grown with or without Ser/Gly or 50 μM cycloheximide (CHX) or 10 μM MG132 for 24 hours. Cells were trypsinized and sorted at the NYU Langone Medical Center Flow Cytometry Laboratory using the MoFlo (Beckman). On the day of sorting, noninfected Ser-coGFPd2 reporter cells were used to establish the 1% sorting gates for the screen (“1% control gate”). Before sorting, 100× representation of postinfected cells were collected for presort samples. Postinfected Ser-coGFPd2 reporter cells were sorted using the 1% control gate until 100× representation was achieved. For each reporter, the screen was performed using two biological replicates that were infected and sorted on different days. Genomic DNA was isolated from sorted cells and prepared for next-generation sequencing (NGS) as previously described (*32*). The CRISPR screen was analyzed using MAGeCK (*69*) and MAGeCKFlute (*70*) to calculate the ranking, β scores, and β score differences. For gene set enrichment analysis, genes with a >0.25 β difference score were used for GO Biological Processes and Molecular Function (MF) using STRINGdb (*71*) and REViGO (*72*).

### Generation of CRISPR KOs

sgRNAs for the indicated genes were cloned into the pLentiCRISPRv2-puromycin plasmid to generate lentivirus using HEK293T cells and lentiviral packaging vectors. Cells were infected with lentiviruses with polybrene (10 μg/ml) for 16 to 24 hours and selected in puromycin (2 μg/ml) for 48 hours. Infected cells expressing sgRNAs were used for downstream experiments at least 7 days after initial lentiviral infection.

### Immunoblotting

Whole cell lysates were generated using modified radioimmunoprecipitation buffer (50 mM tris-HCl (pH 8.0), 150 mM NaCl, 2 mM EDTA, 1% NP-40, and 0.1% SDS, without sodium deoxycholate supplemented with protease (Thermo Fisher Scientific) and phosphatase (10 mM NaF, 1 mM Na_3_VO_4_, 10 mM β-glycerophosphate, and 10 mM sodium pyrophosphate) inhibitors]. Protein lysates were quantified using Bradford Assay (Thermo Fisher Scientific), and equal protein (15-30μg) was loaded into 4-12% gradient Boltz gels (Thermo Fisher Scientific) and transferred with 1× transfer buffer (tris-glycine) and 10 to 15% methanol. Membranes were incubated with the indicated primary antibodies at the manufacturer’s recommendation and visualized with IRDye infrared secondary antibodies, using an Odyssey Infrared imaging system (LI-COR Biosciences). Blots were processed and quantified using Image Studio Lite (LI-COR Biosciences).

### Flow cytometry

GFPd2 and mCherry fluorescence were measured by flow cytometry as previously described (*2*). Briefly, cells were grown with and without Ser/Gly, 50 μM CHX, and/or 10 μM MG132 for 24 hours. Cells were trypsinized, neutralized with defined trypsin inhibitor (Thermo Fisher Scientific), and recovered by centrifugation at 300*g* in a tabletop centrifuge (Beckman Coulter). The samples were resuspended in Hanks’ balanced salt solution + 1% dialyzed FBS and collected using the Yeti ZE5 cell analyzer (Bio-Rad), and the data were analyzed using FlowJo. The geometric mean of Ser-coGFPd2 normalized to mCherry fluorescence was used as a control for expression differences, and the value of CHX-treated cells were subtracted from all the samples to remove the background signal and to calculate the maximum translational effects of Ser/Gly deprivation on coGFPd2 fluorescence. The ratio (−SG/+SG) was calculated using normalized GFPd2 fluorescence from Ser/Gly-deprived cells to Ser/Gly-rich cells.

### Ribosomal profiling

Ribo-seq was performed as described previously (*35*). Cells were washed in cold phosphate-buffered saline (PBS) containing CHX (100 μg/ml) and lysed in 1× polysome lysis buffer [20 mM tris-HCl (pH 7.4), 150 mM NaCl, 5 mM MgCl_2_, 1% Triton X-100, 1 mM dithiothreitol, 10 U of TURBO deoxyribonuclease, CHX (100 μg/ml), and 0.1% NP-40]. Lysates were centrifuged at 4°C at 20,000*g* for 10 min, and supernatants were used to generate ribosome-protected fragments (RPFs).

For RPFs, lysates were digested with RNase I for 45 min at room temperature (RT) and purified with MicroSpin S-400 columns and the Zymo Research RNA clean and Concentrator kit. The RPF footprints were separated on a tris-borate EDTA (TBE)–urea gel, and the 26- to 34-nt RNA bands were excised and extracted for further preparation. T4 Polynucleotide Kinase (T4 PNK) was used for RNA end-repair of RPFs and purified using the Zymo Research RNA clean and Concentrator kit. A 3′ adaptor was ligated to the RPFs using a universal preadenylated linker [New England Biolabs (NEB)], ran on a TBE-urea gel, and purified by gel excision and extraction. SuperScript III (Thermo Fisher Scientific) was used to reversed transcribe ligated RPFs, and cDNAs were excised and extracted from a TBE-urea gel. Next, cDNAs were circularized using circLigase, and rRNA was depleted using a set of biotinylated rRNA depletion oligomers. NGS libraries were amplified from rRNA-depleted cDNAs using Phusion HF, and the products were excised and extracted from a TBE gel. The NGS library was submitted to New York University Langone Medical Center (NYULMC) genome technology core for analysis on the NovaSeq6000.

### Ribo-seq data analysis

Ribo-seq analysis was performed as described by Darnell *et al.* (*3*) and previously described by Banh *et al.* (*2*). Single-end sequencing reads were trimmed for the adapter sequence 5′-CTGTAGGCACCATCAAT-3′ using cutadapt (*73*) with a minimum length of 13 nt. rRNA was removed using bowtie2 (*74*), and the remaining sequences were aligned to the transcriptome using rsem (*75*) and bowtie2. Similarly, the ribosome density at codons was calculated as previously described (*2*, *3*). The average ribosome density around each codon was calculated and normalized to the mean read count for that transcript. For each codon, the average read coverage was found for each position in a 150-nt window on either side of all occurrences of that codon. The change in ribosome density around each codon upon Ser/Gly deprivation was calculated by subtracting the average ribosome density at each position in the 150-nt window around the codon in the Ser/Gly-rich conditions from the Ser/Gly-deprived conditions. This determined the changes in ribosome density on all codons upon Ser/Gly limitation. Similar results were obtained using RiboMiner (*76*).

### RNA sequencing

Total RNA was extracted from cell lines using TRIzol Reagent (Thermo Fisher Scientific,) according to the manufacturer’s protocol. RNA concentration and purity were assessed using a NanoDrop One/OneC Microvolume ultraviolet-visible (UV-Vis) spectrophotometer (Thermo Fisher Scientific). Purified RNA samples were subsequently prepared and analyzed by Novogene Co. Ltd.

### Differential TE calculation

TE was calculated using Xtail (*41*), to determine the differential TE between samples based on RNA-seq and Ribo-seq data. Xtail is available at https://github.com/xryanglab/xtail. Raw read counts from RNA-seq and Ribo-seq libraries were input into Xtail, which then models the relationship between RNA abundance and ribosome occupancy to identify differences in TE. The analysis was performed using default parameters, and transcripts with low read counts were filtered out before TE estimation.

### Reverse transcription quantitative PCR

After total RNA extraction by TRIzol Reagent (Thermo Fisher Scientific), RNA concentration and purity were assessed using a NanoDrop One/OneC Microvolume UV-Vis Spectrophotometer (Thermo Fisher Scientific). Samples underwent reverse transcription using the SuperScript IV VILO Master Mix (Thermo Fisher Scientific) as per the manufacturer’s instructions. For mRNA quantification, we used the SsoAdvanced Universal SYBR Green Supermix (Bio-Rad) and performed the experiments in a QuantStudio5 Thermocycler (Thermo Fisher Scientific). All of the primers were used at a concentration of 500 nM with 18*S* functioning as the housekeeping gene. Relative quantification of gene expression was determined by the 2^–ΔΔCT^ method. Table S4 lists all primers used.

### Charge DM-tRNA-seq and analysis

tRNA charging and fractional abundance measurements were performed as previously described (*39*). TRIzol extracted RNA was treated with 50 mM sodium periodate and repaired with 60 mM sodium borate (pH 9.5), and tRNAs (50 to 150nt) were gel purified and demethylated (ArrayStar tRNA RT-PCR Kit). RNA end-repair was performed using T4 PNK on demethylated tRNAs, purified, ligated to a 3′ universal preadenylated linker (NEB), ran on a TBE-urea gel, and purified by gel excision and extraction. Samples were reversed transcribed using SuperScript III (Thermo Fisher Scientific), and cDNAs were excised and extracted from a TBE-urea gel and circularized using circLigase II. tRNA-seq libraries were amplified using Phusion HF (NEB) and excised and extracted from a TBE gel. tRNA-seq library was submitted to NYULMC genome technology core for analysis on the NextSeq500. Analysis of tRNA charging was performed (*39*) using codes and scripts obtained from T. Pan’s laboratory, which are available at https://github.com/Jessica-Pan/DM-tRNA-seq-ref-genomes. Pre-tRNA and mature tRNA isodecoders were quantified using unique reads from the tRAX pipeline, which is available from T. Lowe’s laboratory (https://trna.ucsc.edu/tRAX/). Isoacceptors were quantified by summing the counts of the respective isodecoders and compared using two-way analysis of variance (ANOVA) followed by two-stage Benjamini-Hochberg correction to determine the adjusted *P* value. Charged tRNAs were quantified by multiplying the percentage charged tRNAs to the mature tRNA reads.

### Fluorescent live-cell imaging

Cells expressing ELAC2–enhanced green fluorescent protein (EGFP) WT or mutants were seeded into glass-bottom black 96-well plates (Cellvis) and allowed to adhere overnight. The next day, the wells were washed with PBS, and cells were grown in phenol red-free DMEM + 10% dialyzed FBS with or without Ser/Gly for 24 hours. The nucleus and mitochondria were stained with Hoechst and MitoTracker DeepRed (Thermo Fisher Scientific), respectively, as per the manufacturer’s instructions. Images were captured using the Cytation C10 (Agilent), a scanning disk confocal microscope. Images were processed and analyzed using the Gen5 software (Agilent).

### Immunofluorescent imaging

Cells were washed in PBS and fixed in 4% methanol-free paraformaldehyde or 10% buffered formalin for 15 min at RT. Fixed cells were permeabilized using 0.1% Triton X-100 in PBS for 10 min at RT and blocked in 4% goat serum for 2 hours at RT or overnight at 4°C. Samples were stained with the indicated primary antibody overnight at 4°C, washed three times in PBS, and stained with anti-mouse or anti-rabbit AlexaFluor PLUS secondary antibodies (Thermo Fisher Scientific) for 1 hour at RT, followed by three washes in PBS and 4′,6-diamidino-2-phenylindole staining with ProLong Gold Antifade (Thermo Fisher Scientific). The samples were imaged using a scanning disk confocal microscope, the Cytation C10 (Agilent). Images were processed and analyzed using the Gen5 software (Agilent). Laser scanning confocal images were taken using a Zeiss LSM 880 laser scanning confocal Microscope and analyzed at the New York University Langone Health (NYULH) microscopy laboratory.

### Exogenous tRNA^Ser^ delivery

Mature tRNA^Ser^ was synthesized by Integrated DNA Technologies as HPLC (high-performance liquid chromatography)–purified RNA Ultramers or in vitro as described (see table S4) (*40*). To fold tRNAs, in vitro–synthesized tRNAs were denatured at 95°C for 2 min, followed by 3 min at 22°C, incubated at 37°C for 5 min, and stored at −80°C (*77*). Exogenous mature tRNA^Ser^ isodecoders were transfected into cells using Lipofectamine RNAiMAX (Thermo Fisher Scientific) as per the manufacturer’s instructions. Briefly, 300 ng of tRNAs was mixed with 0.6 μl of Lipofectamine RNAiMAX and incubated for 15 min at RT before addition into a single 96-well sample. The next day, the media was replaced with DMEM + 10% dialyzed FBS with or without Ser/Gly and CHX for 24 hours. Cells were prepared for flow cytometry analysis on the Yeti ZE5 cell analyzer (Bio-Rad) as described above.

### Stable isotope labeling of amino acids in cell culture

To monitor the levels of newly synthesized proteins, cells were washed in PBS and switched to phenol red-free DMEM without Arg and Lys + 10% dialyzed FBS, supplemented with ^13^C-, ^15^N-Arg–, and -Lys with or without Ser/Gly for 24 hours. Cell pellets were sent to the NYULMC Proteomics Laboratory (New York, NY, USA) for sample preparation, processing, and analysis as described below. Unlabeled peptides represent preexisting proteins synthesized before the addition of ^13^C-, ^15^N-Arg–, and -Lys–containing media. In contrast, ^13^C-, ^15^N-Arg–, and -Lys–labeled peptides correspond to newly synthesized proteins produced within 24 hours following the media change.

### Proteomics

#### Mass spectrometry sample processing

Proteomic samples were prepared, ran, and analyzed with the help of the NYULH proteomics laboratory (New York, NY, USA). Briefly, cell pellets were lysed in 100% trifluoroacetic acid (TFA) for 5 min at RT and subsequently neutralized with 10× (v/v) of 2 M tris containing 10 mM TCEP and 20 mM chloroacetamide for 30 min at 90°C. For enzymatic digestion, lysates were diluted 5× with water containing trypsin (protein:trypsin = 50:1 wt:wt) and incubated overnight at 37°C. Peptide digests were acidified with TFA to final 2%, desalted on C18 StageTips, dried in a SpeedVac, and resolubilized in 0.1% formic acid.

#### Liquid chromatography–coupled mass spectrometry

Liquid chromatography (LC) separation was performed online on EASY-nLC 1000 (Thermo Scientific) using Acclaim PepMap 100 (75 μm by 2 cm) precolumn and PepMap RSLC C18 (2 μm, 100 A by 50 cm) analytical column. Peptides were eluted over 146 min LC gradient from the column directly into an Orbitrap HF-X mass spectrometer (Thermo Fisher Scientific). Data were collected in data-dependent acquisition mode. Mass spectrometry (MS) scans were acquired at resolution 120,000 with AGC set at 3 × 10^−6^ and the maximum injection time of 100 ms. Following each MS scan, up to 20 peptide precursors were isolated (isolation window, 1.4 mass/charge ratio) and fragmented at a normalized collision energy of 27, and fragmentation spectra (tandem MS) were collected at a resolution of 30,000, AGC of 5 × 10^−5^, and a maximum injection time of 100 ms. Dynamic exclusion was set at 45 s. The mass spectrometric raw files are accessible at https://massive.ucsd.edu under accession MSV000097262.

#### MS data analysis

MS data were analyzed using MaxQuant software version 1.6.3.44 and searched against the SwissProt subset of the human UniProt database (http://uniprot.org/). Database search was performed in Andromeda integrated with the MaxQuant environment. A list of 248 common laboratory contaminants in MaxQuant was also added to the database. The enzyme specificity was set to trypsin for searching, with the maximum number of missed cleavages set to 2. Methionine oxidation was searched as a variable modification; carbamidomethylation of cysteine was searched as a fixed modification. The FDR for peptide, protein, and site identification was set to 1%. Subsequent data analysis was performed in either Perseus (http://perseus-framework.org/) or using the R environment for statistical computing and graphics (http://r-project.org/). Scripts for proteomic analysis can be found at https://github.com/Banh-Lab/CostinitiV_2025.

### Binning of Ser-sensitive codons and analysis

RiboMiner was used to obtain the CDSs for the longest transcripts of every gene in the human genome. The CDS length and number of Ser codons for each gene were counted and used to calculate the percentage of AG[U/C], UC[C/U], or UC[G/A] against the total number of Ser codons. Genes were separated into different bins based on the percentage of AG[U/C], UC[C/U], or UC[G/A] over total Ser codons. Using *R* and *dplyr*, a list of genes that overlapped with the proteins identify by SILAC proteomics and RNA-seq was generated to determine their association with the percentage of AG[U/C], UC[C/U], or UC[G/A] against the total number of Ser codons. For binning plots, the average value of each bin was calculated and plotted. DAVID (*78*) (Database for Annotation, Visualization, and Integrated Discovery) also was used to identify enrichment for GO MF proteins in the 61 to 90% bins used to determine the AG[U/C]%- or UC[C/U]%-enriched genes. Canonical pathways with an FDR adjusted *P* value of <0.05 were considered significant.

*z*-Scores for the codon ratio UC[C/U]:UC[C/U] were calculated for human genes as previously described (*2*, *3*). The scripts used for these calculations are available at https://github.com/rasilab/adarnell_2018 (*3*). Because the original *z*-score method did not account for AG[U/C] codons, the same scripts were adapted to calculate *z*-scores for the AG[U/C]:UC[C/U] ratio across human genes. Genes were then grouped into bins based on their computed *z*-scores. Both individual and binned *z*-score datasets were integrated with a proteomic dataset to assess whether *z*-scores could predict protein synthesis outcomes.

Similarly, differential TE values were grouped into bins based on their calculated fold changes. Both individual and binned TE values were integrated with proteomic data and Ser codon percentage bins to evaluate whether TE correlates with protein synthesis outcomes and whether Ser codon usage can predict TE changes.

All comparisons were manually curated and filtered using Microsoft Excel. Graphs were generated, and statistical analyses were performed using GraphPad Prism version 10. When applicable, nonlinear regression curves were fitted, and Spearman correlation coefficients were calculated to assess the strength and direction of associations.

### Quantification and statistical analysis

Sample sizes (*n*), where “*n*” represents definition of center, dispersion and precision measures, and statistical tests for each experiment, are denoted in the figure legends. Each immunoblot was performed at least three times. All MS data represent *n* = 3 biological replicates for each group. All immunofluorescent images show samples, and quantification was performed on all biological replicates in the experiment, with the number of data points indicating the number of independent biological replicates. The between-group variances were similar, and the data were normally distributed. All analyses and graphs were generated with GraphPad Prism 10. A *P* value of <0.05 was considered significant, and precise *P* values can be found in the figures.
