# Supplementary material for: Serine tRNAs compete to regulate the mRNA translation of serine-sensitive codons
Source: Sci Adv. 2025 Nov 14;11(46):eady4521. doi: 10.1126/sciadv.ady4521 (PMC12617527; doi:10.1126/sciadv.ady4521)
Supplement: Supplementary file 1 — Figs. S1 to S5 Legends for tables S1 to S4 [file sciadv.ady4521_sm.pdf]

Science Advances
AAAS

# Supplementary Materials for

## Serine tRNAs compete to regulate the mRNA translation of serine-sensitive codons

Veronica Costiniti *et al.*

Corresponding author: Robert S. Banh, robert.banh@nyulangone.org;
Alec C. Kimmelman, alec.kimmelman@nyulangone.org

*Sci. Adv.* **11**, eady4521 (2025)
DOI: 10.1126/sciadv.ady4521

**The PDF file includes:**

Figs. S1 to S5
Legends for tables S1 to S4

**Other Supplementary Material for this manuscript includes the following:**

Tables S1 to S4

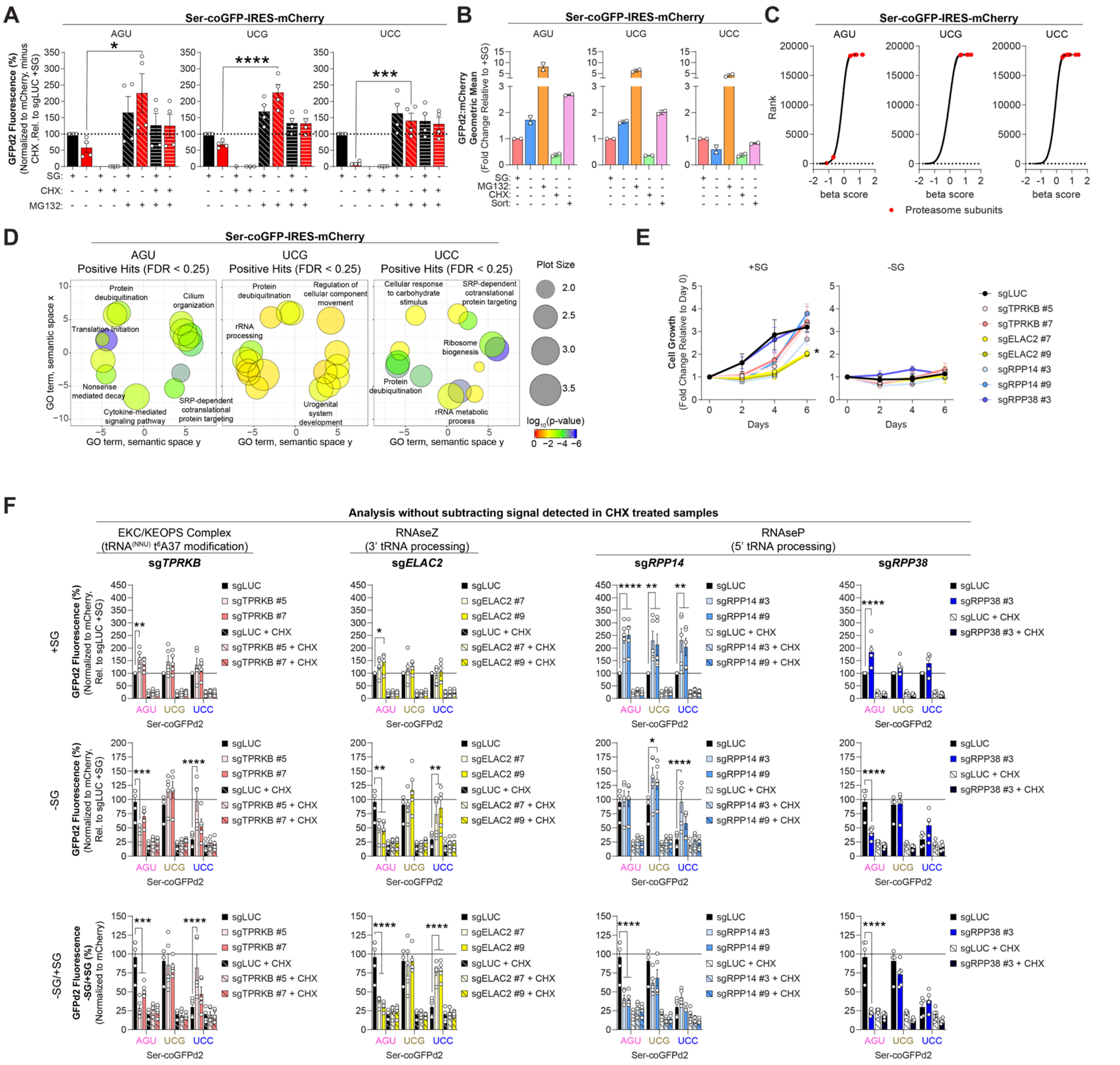

# Fig. S1. CRISPR knockout (KO) screen using Ser-coGFPd2 reporter cells. Related to Figure 1.

**A**, Quantification of Ser-coGFPd2 fluorescence (-SG/+SG) ratios in PATU-8902 reporter cells grown in the presence or absence of Ser/Gly (SG), MG132 (proteasomal inhibitor), or CHX (cycloheximide) for 24 hours. Note that proteasomal inhibition prevents decrease in Ser-coGFPd2 fluorescence. (*n=4*).

**B**, Geometric mean of GFPd2:mCherry signal from Ser-coGFPd2 reporter expressed in PATU-8902 cells grown with or without Ser/Gly (SG), MG132 (proteasomal inhibitor), and cycloheximide (CHX) for 24 hours (from **Fig.1B**). Similarly, cells infected with the whole-genome sgRNA CRISPR library were measured and sorted. (*n*=2).

**C**, Beta scores calculated from whole genome CRISPR KO screen of PATU-8902 Ser-coGFPd2 reporter cells (from **Fig.1B**) grown in the absence of Ser/Gly. Proteasomal subunits are highlighted in red as common positive controls in all the indicated reporter lines.
**D**, Revigo plots of the GO terms from the top positive hits (false discovery rate, FDR <0.25) identified from the CRISPR KO screen (from **Fig.1B**).
**E**, Growth curves of PATU-8902 cells expressing sg*TPRKB*, sg*ELAC2,* sg*RPP14*, or sg*RPP38* grown in Ser/Gly-rich or -deprived conditions. Cellular proliferation remains suppressed in the absence of Ser/Gly, regardless of sgRNA expression. (*n*=3).
**F**, Quantification of GFPd2 fluorescence in the presence (+SG) or absence (-SG) of Ser/Gly, or ratio (-SG/+SG) (related to **Fig.2C** and **Fig.S2A**). PATU-8902 Ser-coGFPd2 reporter cells expressing sgLUC (control), sg*TPRKB*, sg*ELAC2*, sg*RPP14*, and sg*RPP38* were grown in the presence or absence of Ser/Gly (SG), or CHX (cycloheximide) for 24 hours. GFPd2 fluorescence was normalized to mCherry and the signal detected in CHX treatment was not subtracted. (*n=5*).
*"n"* represents the number of biologically independent replicates. Graphs (mean ± s.e.m.) were compared using one-way ANOVA (**A**) or two-way ANOVA (**E**, **F**), followed by Tukey post-hoc test (**p<0.05, **p<0.01, ***p<0.005, ****p<0.0001*).

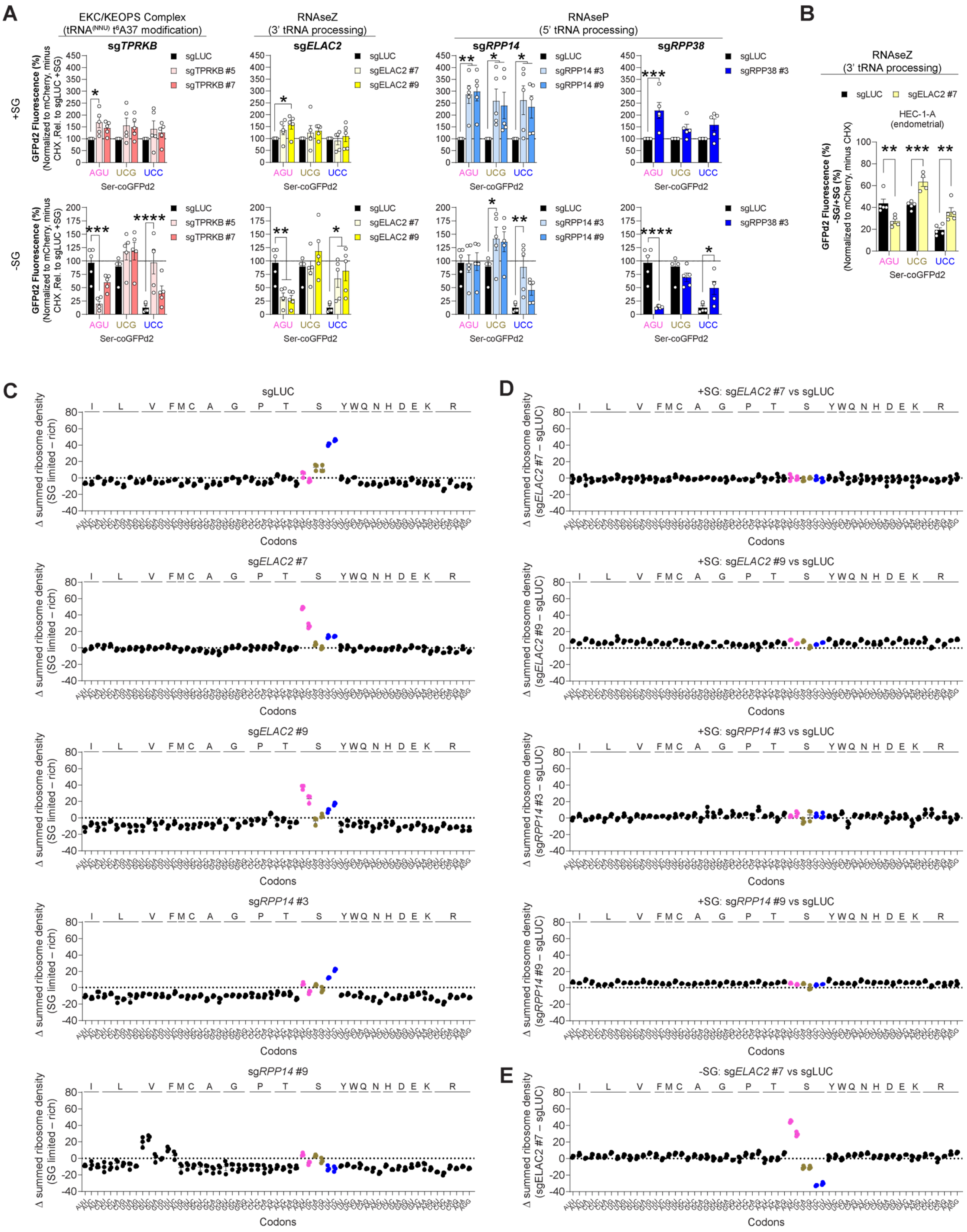


**Fig. S2. Genetic validation of genes mTE regulators of Ser-sensitive codons. Related to Figure 2.**

**A**, Quantification of GFPd2 fluorescence from the indicated PATU-8902 Ser-coGFPd2 reporter cells expressing sgLUC (control), sg*TPRKB,* sg*ELAC2*, sg*RPP14*, and sg*RPP38*. Cells were grown in the presence or absence of Ser/Gly (SG), or CHX (cycloheximide) for 24 hours. GFPd2 fluorescence was normalized to mCherry and CHX treatment. (*n=5*).
**B**, GFPd2 fluorescence (-SG/+SG) ratios from HEC-1-A (endometrial) Ser-coGFPd2 reporter cells expressing sgLUC (control) or sgELAC2 #7. Cells were grown with or without Ser/Gly or CHX for 24 hours. GFPd2 fluorescence was normalized to mCherry and CHX treatment. (*n=4-6*).
**C**, Extension of the ribosomal densities (from **Fig.2D**) for all codons in mRNA in Ser/Gly-deprived conditions for the indicated cells. Ser/Gly-limitation only increases the ribosomal densities around Ser, but not other amino acid codons. (*n=3*).
**D**, Effects of *ELAC2*- or *RPP14*-KO on the ribosomal densities in Ser/Gly-rich conditions compared to PATU-8902 control (sgLUC) cells. Note that *ELAC2*- or *RPP14*-KO does not affect ribosomal densities on any codons in Ser/Gly-rich conditions. (*n=3*).
**E**, Highlighting differences of ribosomal densities of PATU-8902 *ELAC2*-KO compared to control (sgLUC) cells grown in Ser/Gly-poor conditions. Note that *ELAC2*-KO switches the Ser-sensitive codons from UC[C/U] to AG[U/C] in Ser/Gly-poor environments. (*n=3*).
Where "*n*" represents the number of biologically independent experiments for each group and condition. Graphs (mean ± s.e.m.) were compared using two-way ANOVA (**A**-**B**), followed by Bonferroni post-hoc test (*$*p<0.05$, $**p<0.01$, $***p<0.005$, $****p<0.0001$*).

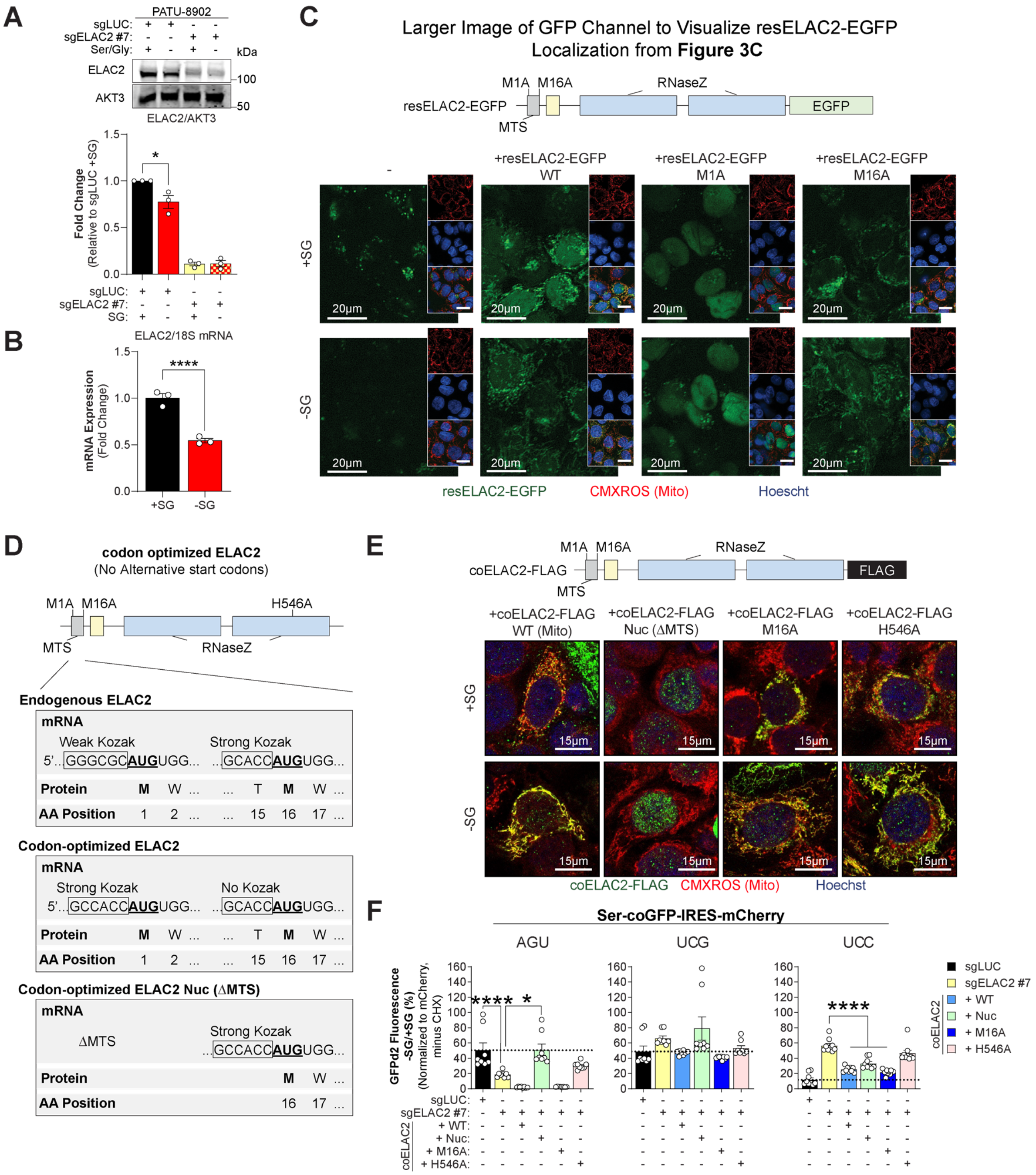


**Fig. S3. Codon-optimized ELAC2 drives mitochondria localization and does not restore mTE of AG[U/C] Ser codons. Related to Figure 3.**

**A**, Immunoblot and quantification of endogenous ELAC2 protein levels in PATU-8902 control and *ELAC2*-KO cells grown in Ser/Gly-rich and -poor conditions for 24 hours. AKT3 serves as a loading control. (*n=3*).

**B**, mRNA expression of endogenous ELAC2 in PATU-8902 control and *ELAC2*-KO cells grown in Ser/Gly-rich and -poor conditions for 24 hours. (*n=3*).

**C**, Enlarged EGFP channel insets of the same images found in **Fig.3C.** Schematic of resELAC2 with a C-terminal EGFP tag. The mitochondria and nucleus were stained using MitoTracker DeepRed and Hoechst, respectively. Scale bar is shown.

**D**, Schematic of ELAC2 domains, signal sequences, and mutations used for codon-optimized (co)-ELAC2. Codon optimization places a strong Kozak sequence at the first AUG start codon and destroys the Kozak sequence in the second start codon. The mitochondrial targeting sequence (MTS) was removed to drive expression of coELAC2 to the nucleus (ELAC2 Nuc).

**E**, Representative immunostaining of coELAC2-FLAG WT or mutants in Ser/Gly-rich or -poor environments. The mitochondria and nucleus were stained using CMXROS and Hoechst, respectively. coELAC2-FLAG WT, M16A or H546A mainly localizes to the mitochondria. While coELAC2-FLAG Nuc mutant localizes to the nucleus. Scale bar is shown.

**F**, Ser-coGFPd2 fluorescence (-SG/+SG) ratios in the indicated PATU-8902 reporter cells in response to Ser/Gly-deprivation. coGFPd2 fluorescence was normalized to mCherry and CHX signal was subtracted to remove background signal. Mitochondrial coELAC2 (e.g. WT or M16A) completely suppresses and does not rescue the mTE of AGU-coGFPd2 fluorescence in *ELAC2*-KO cells deprived of Ser/Gly. ($n=8$).

Where "*n*" represents the number of biologically independent experiments for each group and condition. Graphs (mean ± s.e.m.) were compared using a Student t-test (**B**) or one-way ANOVA (**A, F**), followed by Bonferroni's post-hoc test ($*p<0.05$, $**p<0.01$, $***p<0.005$, $****p<0.0001$).

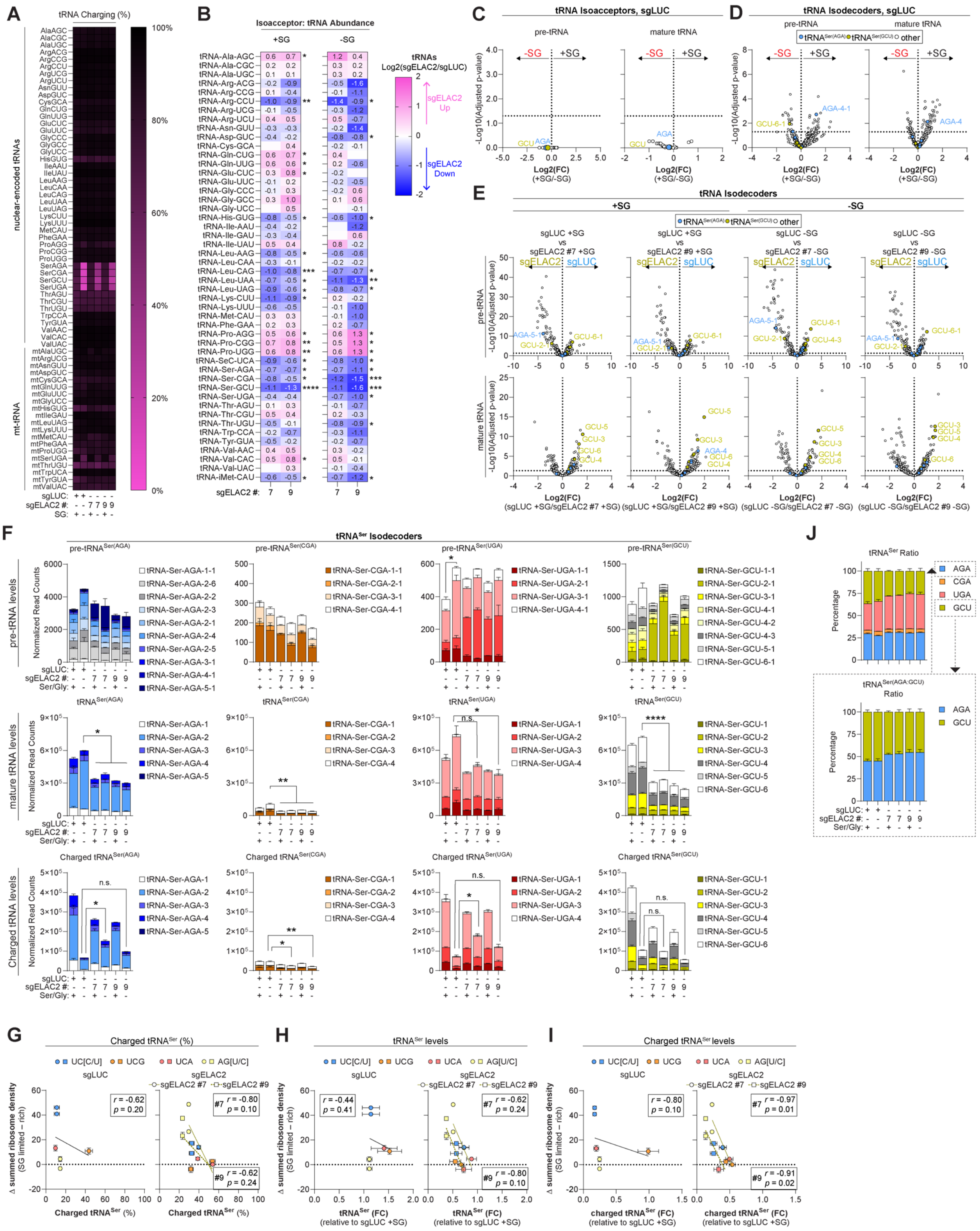


**Fig. S4. ELAC2 deletion alters the tRNA pools in cells. Related to Figure 4.**

**A**, Heatmap of tRNA charging for all tRNA isoacceptors from the PATU-8902 control (sgLUC) and *ELAC2*-KO cells grown with or without Ser/Gly (SG) for 24 hours. (*n=3*).

**B**, Quantification of the differences in tRNA levels due to *ELAC2*-KO in PATU-8902 cells grown in Ser/Gly-rich and -poor conditions. Heatmaps represent the mean value. (*n=3*).
**C-D**, Premature (pre)- and mature tRNA isoacceptor (**C**) or isodecoder (**D**) differences in PATU-8902 control (sgLUC) cells grown in the presence or absence of Ser/Gly (SG) for 24 hours. $tRNA^{Ser(AGA)}$ and $tRNA^{Ser(GCU)}$ are highlighted. (*n=3*).
**E**, Fold change differences of pre- and mature-tRNA isodecoders of PATU-8902 control (sgLUC) and *ELAC2*-KO cells grown in the absence of Ser/Gly for 24 hours. $tRNA^{Ser(AGA)}$ and $tRNA^{Ser(GCU)}$ isodecoders are highlighted. (*n=3*).
**F**, Detailed level differences of pre-, mature, and charged $tRNA^{Ser}$ isodecoders in the PATU-8902 control or *ELAC2*-KO cells grown with or without Ser/Gly for 24 hours. (*n=3*).
**G-I**, Correlation of ribosome densities in relation to the respective percent $tRNA^{Ser}$ charging (**G**), mature tRNA (**H**) or charged tRNA (**I**) isodecoders from the indicated PATU-8902 cells deprived of Ser/Gly relative to sgLUC cells grown in Ser/Gly-rich conditions. The Spearman correlation (*r*) and *p*-value are as shown. (*n=3*).
**J**, Ratios of total levels of $tRNA^{Ser}$ isoacceptors in PATU-8902 control and *ELAC2*-KO cells grown in Ser/Gly-rich and -poor conditions for 24 hours. Isolated comparison of $tRNA^{Ser(AGA)}$ and $tRNA^{Ser(GCU)}$ isoacceptor ratios shown at the bottom panel. (*n=3*).
Arrow direction indicates higher protein abundance in control or *ELAC2*-KO cells as indicated. Dotted line on the y-axis represents adjusted *p*-value = 0.05 (**C-E**). Where *"n"* represents the number of biologically independent experiments for each group and condition. Graphs represent mean ± s.e.m. and were compared using one-way ANOVA (**F, J**) or two-way ANOVA (**B-E**), followed by Tukey (**J**), Dunnett's (**F**) post-hoc test or Two-stage Benjamini–Hochberg correction (**B-E**). (*$^*p<0.05$, $^{**}p<0.01$, $^{***}p<0.005$, $^{****}p<0.0001$*).

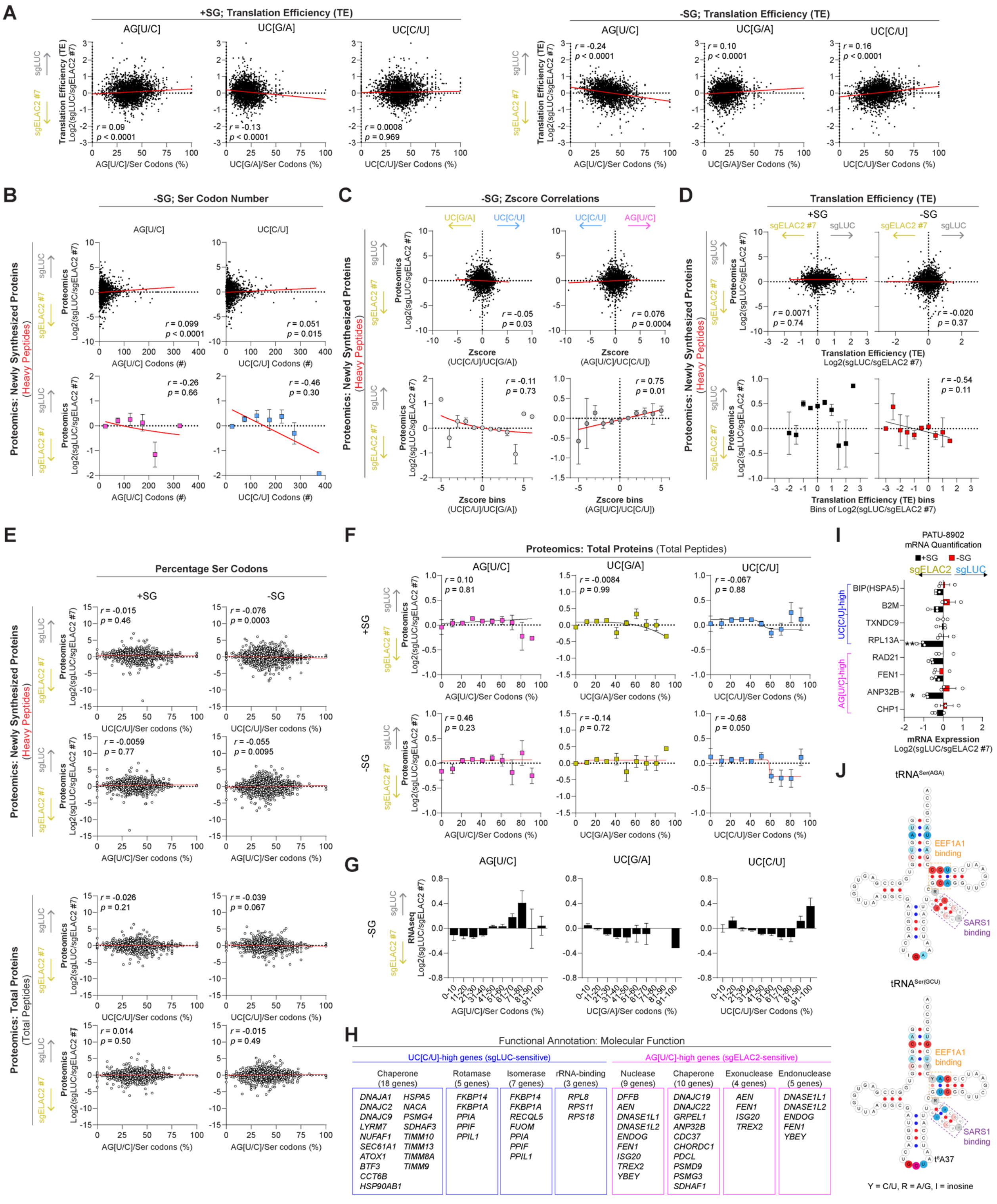


**Fig. S5. Ser-sensitive codons affect the proteome of newly synthesized proteins in Ser/Gly-poor environments. Related to Figure 5.**

**A**, Fold change of individual calculated translation efficiency (TE) between PATU-8902 control (sgLUC) and *ELAC2*-KO cells cultured in Ser/Gly-rich (+SG) and poor media (-SG) for 24 hours, plotted relative to the percentage of the indicated Ser codon for that gene.
**B**, For Ser/Gly-deprived samples, scatter plot of the differences in newly synthesized proteins (heavy peptides) against the absolute number of the indicated Ser codons plotted individually per gene (Top). For binned analyses, average differences in newly synthesized proteins were plotted (Bottom).
**C**, Scatter plots of the differences in newly synthesized proteins (heavy peptides) plotted against the corresponding gene-specific Z-score calculations (Top) in Ser/Gly-poor conditions. Scatter plots showing the average differences in newly synthesized proteins plotted against gene-specific Z-scores within each bin (Bottom) in Ser/Gly-poor conditions.
**D**, Scatter plots of the differences in newly synthesized proteins (heavy peptides) plotted against the corresponding TE differences between PATU-8902 control and *ELAC2*-KO cells grown in Ser/Gly-rich or -poor conditions (Top). Scatter plots showing the average differences (mean ± s.e.m.) in newly synthesized proteins (heavy SILAC peptides) plotted against TE differences within each bin (Bottom).
**E**, Correlation plots of percentage bins of Ser-sensitive codons relative to newly synthesized (Top) or total (Bottom) protein fold change differences of the indicated cells grown in Ser/Gly (SG)-rich or -poor conditions for 24 hours. (*n=3*).
**F**, Fold change differences of total proteins in PATU-8902 control (sgLUC) and *ELAC2*-KO cells grown with or without Ser for 24 hours relative to the percentage bin of the indicated Ser codon over the total Ser codons. A non-linear regression line was fitted that excluded outliers, and Spearman correlation coefficients are represented by *r*. (*n=3*).
**G**, Histogram of the RNA-seq fold change differences between PATU-8902 control (sgLUC) and *ELAC2*-KO cells grown without Ser/Gly for 24 hours for the indicated percentage bin of Ser codon over the total Ser codons. (*n=3*).
**H**, UC[C/U]- or AG[U/C]-high genes enriched in functional annotation of molecular function are shown in blue and pink, respectively.
**I**, Quantification of mRNA to determine ELAC2-dependent changes in mRNA expression of UC[C/U]- and AG[U/C]-high genes in Ser/Gly-replete and -deprived conditions. Related to **Fig.5F**. (*n=3*).
**J**, Schematic comparing the differences of $tRNA^{Ser(AGA)}$ and $tRNA^{Ser(GCU)}$ isoacceptors. A-U and G-C bonds are highlighted in blue and red, respectively. Complete differences (e.g. A-U vs G-C) at the same position are marked in solid colors, while mirrored changes (e.g. A-U vs U-A) are shown in faded colors. The variable arm is important for Ser-tRNA synthetase (SARS1) binding, while the T-stem is important for EEF1A1 binding. Arrow direction indicates higher protein abundance in control or *ELAC2*-KO cells as indicated. The Spearman correlation (*r*) and *p*-value are as shown (**A-F**). Where *"n"* presents the number of biologically independent replicates. Graphs (mean ± s.e.m.) were compared using two-way ANOVA (**I**), followed by Dunnett's post-hoc test. (*$*p<0.05$, $**p<0.01$, $***p<0.005$, $****p<0.0001$*).

**Table S1.**

Ser codon sgRNA CRISPR Screen. Calculated beta scores and beta diff for CRISPR screen using Ser-coGFPd2 reporters

**Table S2.**

OMICS datasets. SILAC proteomics, RNA-seq, and CDS codon information.

**Table S3.**

Protein enrichment analysis of Ser-sensitive genes. SILAC proteomics, RNA-seq, and CDS codon information.

**Table S4.**

List of reagents used in study.
